# Natural Anticancer Peptides from Marine Animal Species: Evidence from In Vitro Cell Model Systems

**DOI:** 10.3390/cancers16010036

**Published:** 2023-12-20

**Authors:** Mariangela Librizzi, Chiara Martino, Manuela Mauro, Giulia Abruscato, Vincenzo Arizza, Mirella Vazzana, Claudio Luparello

**Affiliations:** 1 Dipartimento di Scienze e Tecnologie Biologiche Chimiche e Farmaceutiche (STEBICEF), Università di Palermo, 90128 Palermo, Italy; librizzimariangela87@gmail.com (M.L.); chiara.martino@unipa.it (C.M.); manuela.mauro01@unipa.it (M.M.); vincenzo.arizza@unipa.it (V.A.); mirella.vazzana@unipa.it (M.V.); 2 National Biodiversity Future Center (NBFC), 90133 Palermo, Italy

**Keywords:** marine drugs, anticancer peptides, tumor cells, Porifera, Cnidaria, Mollusca, Annelida, Arthropoda, Echinodermata, Chordata

## Abstract

**Simple Summary:**

Anticancer peptides are short aminoacidic chains, which display selective cytotoxicity mostly against tumor, but not healthy, cells through interference with intracellular biological events. The marine environment features an ever-growing level of biodiversity and, therefore, seas and oceans are indeed poorly exploited mines in terms of natural products of biomedical interest. Adaptation processes to extreme and competitive environmental conditions led marine species to produce unique metabolites, which have found broad use for various applications in healthcare management, due to their anticancer, anti-angiogenic, anti-inflammatory and regeneration abilities. The aim of this review is to pick and list selected studies that report on the isolation of marine animal-derived peptides and the identification of their anticancer activity in in vitro cultures of cancer cells.

**Abstract:**

Anticancer peptides are short and structurally heterogeneous aminoacidic chains, which display selective cytotoxicity mostly against tumor cells, but not healthy cells, based on their different cell surface properties. Their anti-tumoral activity is carried out through interference with intracellular homeostasis, such as plasmalemma integrity, cell cycle control, enzymatic activities and mitochondrial functions, ultimately acting as angiogenesis-, drug resistance- and metastasis-inhibiting agents, immune stimulators, differentiation inducers and necrosis or extrinsic/intrinsic apoptosis promoters. The marine environment features an ever-growing level of biodiversity, and seas and oceans are poorly exploited mines in terms of natural products of biomedical interest. Adaptation processes to extreme and competitive environmental conditions led marine species to produce unique metabolites as a chemical strategy to allow inter-individual signalization and ensure survival against predators, infectious agents or UV radiation. These natural metabolites have found broad use in various applications in healthcare management, due to their anticancer, anti-angiogenic, anti-inflammatory and regeneration abilities. The aim of this review is to pick selected studies that report on the isolation of marine animal-derived peptides and the identification of their anticancer activity in in vitro cultures of cancer cells, and list them with respect to the taxonomical hierarchy of the source organism.

## 1. A Brief Insight into Anticancer Peptides

Peptides, i.e., aminoacidic chains linked by covalent bonds and ranging between 10 and 100 monomers, exert very disparate biological effects in cells and tissues, influencing a multitude of biochemical functions in the body. A group of such molecules, which are found across all organisms and are typically cationic, amphiphilic and rich in hydrophobic residues, have been shown to play a role within the innate immunity system, possessing antimicrobial properties due to their ability to interact with the negatively charged membrane of microorganisms and induce cell damage and death. Antimicrobial peptides have led to the discovery of anticancer peptides, a subset which displays selective cytotoxicity mostly against tumor, but not healthy, cells based on their different cell surface properties [1]. These peptides are heterogeneous and endowed with different secondary structures allowing them to be grouped into four categories as follows: α-helical, β-pleated sheets, random coil and cyclic [2]. Their anticancer activity is carried out through interference with intracellular homeostasis, such as plasmalemma integrity, cell cycle control, enzymatic activities and mitochondrial functions, ultimately acting as angiogenesis-, drug resistance- and metastasis-inhibiting agents, immune stimulators, differentiation inducers and necrosis or extrinsic/intrinsic apoptosis promoters [3,4].

## 2. Bioactive Molecules from Marine Organisms

The marine environment, which covers three-quarters of the Earth’s surface, is the largest habitat on the globe. It features an ever-growing level of biodiversity linked to the variety of environments, with the consequence that numerous new marine species are continuously discovered every year. Therefore, seas and oceans are indeed poorly exploited mines in terms of bioactive natural products, and marine biotechnology and pharmacology represent constantly developing key themes aimed at increasing the utilization of marine natural resources [5]. In the course of adaptation processes to extreme and competitive environmental conditions, marine species, especially if sessile, met with the promotion and accumulation of (epi)genotypic and phenotypic changes, leading to the production of a plethora of unique secondary metabolites as a chemical strategy to allow inter-individual signalization and ensure survival against predators, infectious agents or UV radiation [6,7]. These natural metabolites, whose chemical communication ability encompasses different taxonomic lineages and even kingdoms, have found broad use in various applications, such as healthcare management, biomaterial manufacturing and environmental remediation, and, due to their peculiar chemical structures and properties, also in the development of new classes of molecules through the design of analogs with greater bioavailability and efficacy and less toxicity. To cite a few examples, this is the case for extracts, or isolated primary and secondary metabolites, obtained from marine vertebrates and invertebrates and seaweeds, which have shown anticancer, anti-angiogenic, anti-inflammatory and wound healing/skin regeneration abilities [8,9,10,11,12,13,14,15,16,17,18,19]. Moreover, the processing of by-products from marine animals and plants has also granted a number of beneficial properties due to their abundance in polyunsaturated fatty acids, amino acids, chitosan, carotenoids and polyphenols, among others [20,21].

In consideration of the above, the aim of this review is to pick selected studies that report on the isolation of marine animal-derived peptides and the identification of their anticancer activity in in vitro cultures of cancer cells, and list them with respect to the taxonomical hierarchy of the source organism. 

## 3. Review Methodology

The methodology used in this review involved the consultation of the PubMed, Scopus and Web of Science databases, and the utilization of relevant keywords, such as “anticancer”, “antitumoral”, “peptides”, “marine”, “natural”, followed by a comprehensive reading of the literature on the selected molecules. The inclusion criteria were the model studied (i.e., in vitro) and the relevance to the topic, including the in-depth analysis of the molecular mechanisms, signaling pathways and action targets of the peptides. Studies that reported data on uncharacterized peptide mixtures, or peptide cytotoxicity in terms of the sole evaluation of IC_50_ on single or panels of cell lines, as well as those dealing with recombinant or chemically synthesized and subsequently modified peptides were excluded. The extracted data were analyzed and synthesized to provide a comprehensive overview of the state-of-the-art on the subject matter.

## 4. Marine Animals as Sources of Anticancer Peptides: In Vitro Evidence

### 4.1. Porifera—Demospongiae

**Figure 1:**
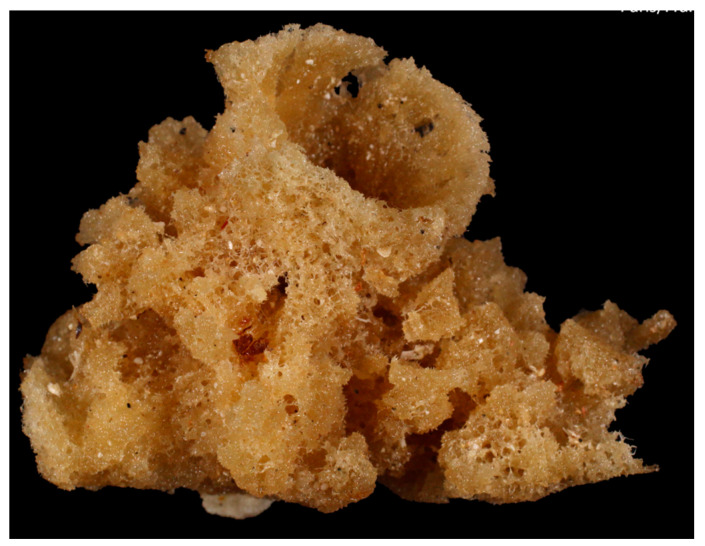
Specimen of *C. basilana*. Credit: RECOLNAT (ANR-11-INBS-0004)—Marie Hennion—(CC-BY 4.0). http://mediaphoto.mnhn.fr/media/1457603880888AD4dw03lT3Rng1tE (accessed on 6 March 2023).

*Clathria basilana* (Lévi, 1961; Porifera, Demospongiae, Poecilosclerida, Microcionidae; Figure 1), a.k.a. red vase sponge, is a sessile tropical species with a typically light red color, tending towards light plum, distributed in the Indo-West Pacific, Palawan/North Borneo and Banda Sea areas [22,23].

**Figure 2:**
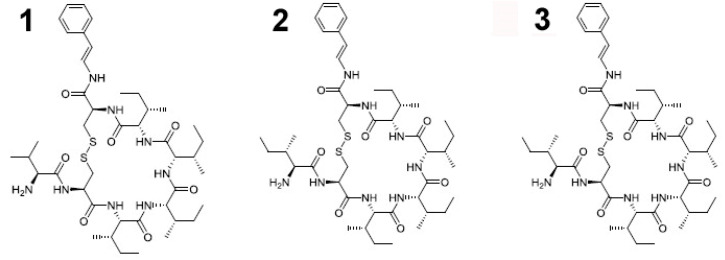
Structures of microcionamide A (**1**), C (**2**) and D (**3**) [24].

In 2017, Mokhlesi et al. [24] demonstrated the strong cytotoxic effect (IC_50_ values ranging from 0.45 to 28 μM) exerted by the peptides microcionamides A, C and D (Figure 2), obtained from this demosponge, against different cancer cells, i.e., Ramos lymphoma, HL-60, Nomo-1 and Jurkat J16 leukemia and A2780 ovarian carcinoma cell lines. Further biological investigation showed that the three compounds induced apoptotic death in Jurkat J16 and Ramos cells. In particular, they promoted the fast activation of caspase-3 in both cell lines with rapid kinetics, although microcionamide D was active at a 10-fold higher concentration (10 μM vs. 1 μM concentration of microcionamides A and C). Moreover, microcionamide A and C were proven to block the starvation-induced degradation of LC3, a key autophagosomal component, in murine embryonic fibroblasts stably expressing mCitrine-hLC3B, thereby suggesting the impairment of the pro-survival signaling of cancer cells, also via the inhibition of autophagy.

**Figure 3:**
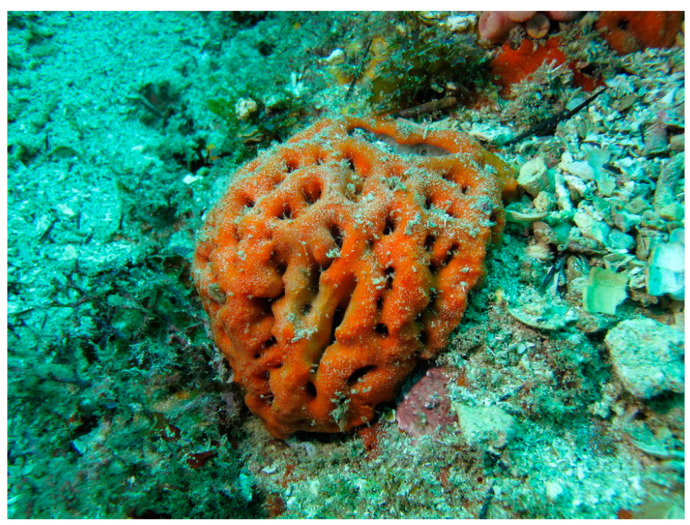
Specimen of *G. corticostylifera*. Author: Eduardo Hajdu (CC BY-NC-SA 4.0). https://www.marinespecies.org/porifera/porifera.php?p=image&tid=191313&pic=148142 (accessed on 6 March 2023).

*Geodia corticostylifera* (Hajdu, Muricy, Custodio, Russo and Peixinho, 1992; Porifera, Demospongiae, Tetractinellida, Geodiidae; Figure 3), to date accepted as *Geodia tylastra*, is a demosponge, typically distributed along Brazil coasts. Its external color tends towards orange, sometimes reddish, whereas the internal color is cream to beige; its shape is globular, cerebriform, up to 12 cm high and 25 cm wide, with a slightly hispid and wrinkled surface [25].

**Figure 4:**
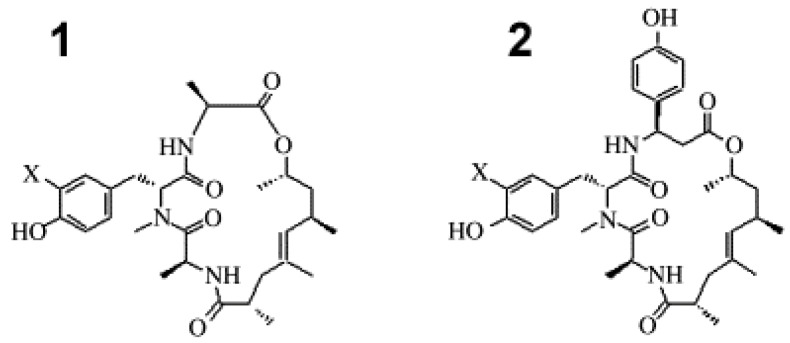
Structures of geodiamolides A and B (**1**, X = I and Br, respectively), and geodiamolides H and I (**2**, X = I and Br, respectively) [26].

The cyclic peptides, geodiamolides A, B, H and I (Figure 4), were isolated from *G. corticostylifera* and their anti-proliferative effects were tested on human breast cancer cell lines T47D and MCF7 [26]. The obtained values of EC_50_ for all the compounds were in the nM range; being geodiamolides, A/H and B/I were more effective on T47D and MCF7 cells, respectively. Using confocal fluorescence microscopy, geodiamolides A, B, H and I were proven to operate in a dose-dependent manner through actin filament disorganization and gathering in the cytoplasm, with consequent displacement of the nuclei from the central position and shape alteration. Interestingly, normal cell lines, i.e., primary culture human fibroblasts and BRL3A rat liver epithelial cells, were not affected by the treatment with geodiamolide H and only the fibroblasts were weakly affected by geodiamolide A, thus suggesting the beneficial biomedical potential of these compounds.

Subsequently, Freitas et al. [27] investigated the effect of geodiamolide H on spheroids obtained from MCF10A normal mammary epithelial cells and the, respectively, non-invasive and invasive/metastatic MCF7 and Hs578T breast tumor cell lines. The compound was proven not to affect the overall morphology and actin organization of MCF10A and MCF7 cells, while inducing cytoskeletal alterations and a reversion of the malignant phenotype in Hs578T cells, which displayed a decreased migratory and invasive ability in vitro, as evidenced by time-lapse video microscopy and Boyden chamber assays. In addition, geodiamolides H and, to a lesser extent, A were found to increase the length of gap junction plaques in rat hepatocarcinoma cells in the absence of actin filament disorganization, due to the improvement of the delivery pathway of the connexin-43 protein [28].

**Figure 5:**
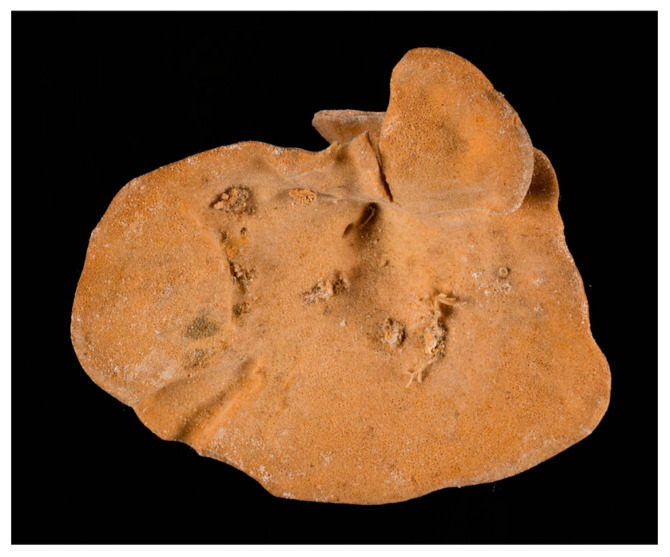
Specimen of *C. lamellata*, an example of *Cymbastela* sp. (CC.BY.4.0). https://en.wikipedia.org/wiki/Cymbastela_lamellata#/media/File:Cymbastela_tricalyciformis_(Bergquist,_1970)_(AM_MA36086-1).jpg (accessed on 16 January 2023).

*Cymbastela* sp. (Hooper and Bergquist, 1992; Porifera, Demospongiae, Axinellida, Axinellidae; Figure 5) is a genus of lamellate coral reef demosponges, which differ in regard to their spicule geometry and length, axial and extra-axial skeletal development, growth form, lamella thickness and live color.

**Figure 6:**
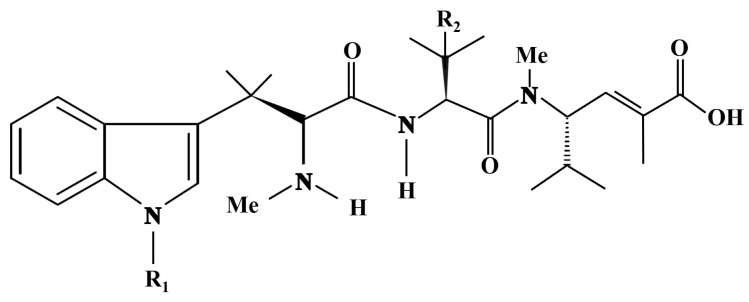
Structure of hemiasterlins. Hemiasterlin: R_1_ and R_2_ = Me; hemiasterlin A: R_1_ = H, R_2_ = Me; hemiasterlin B: R_1_ and R_2_ = H. Redrawn from [29].

Hemiasterlin, hemiasterlin A and hemiasterlin B (Figure 6) are cytotoxic tripeptides isolated from this demosponge species, which act as potential antitumor drugs. Anderson et al. [29] exposed MCF-7 human breast cancer cells to the IC_50_ of the compounds, ranging from 0.5 to 7 nM, and identified their role as microtubule inhibitors causing mitotic arrest through the derangement of the cytoskeletal dynamics and, therefore, being potential chemotherapeutic agents. Further biochemical studies demonstrated that hemiasterlin inhibited, in a non-competitive manner, the binding of vinblastine to tubulin, stabilized the colchicine-binding activity of tubulin, inhibited the nucleotide exchange on β-tubulin and induced the formation of ring-like tubulin oligomers [30]. More recently, analogs of hemiasterlin endowed with higher accessibility and potency have been entered in clinical trials [31,32].

### 4.2. Cnidaria—Antozoa

**Figure 7:**
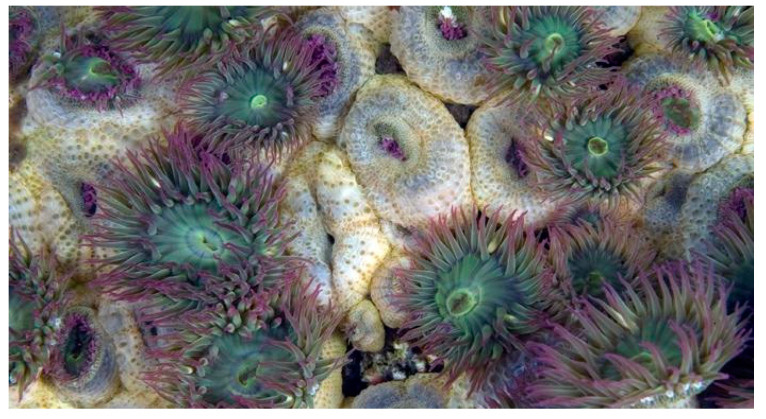
Specimen of *A. elegantissima*. Author: Neil McDaniel (CC BY-NC-SA 4.0). https://www.marinespecies.org/aphia.php?p=image&tid=283347&pic=117079 (accessed on 14 March 2023).

*Anthopleura elegantissima* (Brandt, 1835; Cnidaria, Anthozoa, Actiniaria, Actiniidae; Figure 7), also known as the aggregating anemone or clonal anemone, is a very abundant species forming clonal aggregations of polyps on the rocky shores along the Pacific coast of North America. This organism is known to host the endosymbiotic algae zooxanthellae, thereby being a model organism for the study of symbioses in cnidarian species [33]. It is highly competitive for space and characterized by the ability to emit poison using structures of “aggression” called acrorhagi [34].

The voltage-gated potassium channel human ether-à-go-go 1 (hEag1, K_V_10.1), undetectable in normal tissues except for central nervous tissue, is widely overexpressed in different human tumor cyto- and histotypes, thereby being considered as a potential target for anticancer treatment [35,36]. In search of novel K_V_10.1 inhibitors, Moreels et al. [37] reported the isolation of the peptide APETx4 (GTTCYCGKYIGIYWFGKYSCPTNRGYTGSCPYFLGICCYPVD) from *A. elegantissima,* which is able to bind to closed K_V_10.1 channels through its YFL hydrophobic patch and the charged residues present on one side and reduce their activation rate. When APETx4 was tested on various human cell models in vitro, it was found to induce a concentration-dependent cytotoxic and apoptosis-promoting effect on neuroblastoma SH-SY5Y, prostate LNCaP, melanoma MDA-MB-435S and epithelial hTERT RPE-1 lines, through its binding to K_V_10.1 and/or other membrane targets, since the peptide resulted in being not very selective. More recent studies have been directed to reveal the APETx4–K_V_10.1 interactions in an atomic resolution, using protein docking and multiscale molecular dynamics techniques. The results obtained have shown that APETx4 is endowed with multiple binding sites and its inhibitory effect is likely due to induced steric effects that prevent the contact of the extracellular loop in the channel with its voltage sensor domain, thus stabilizing the channel structure in the deep closed state [38].

**Figure 8:**
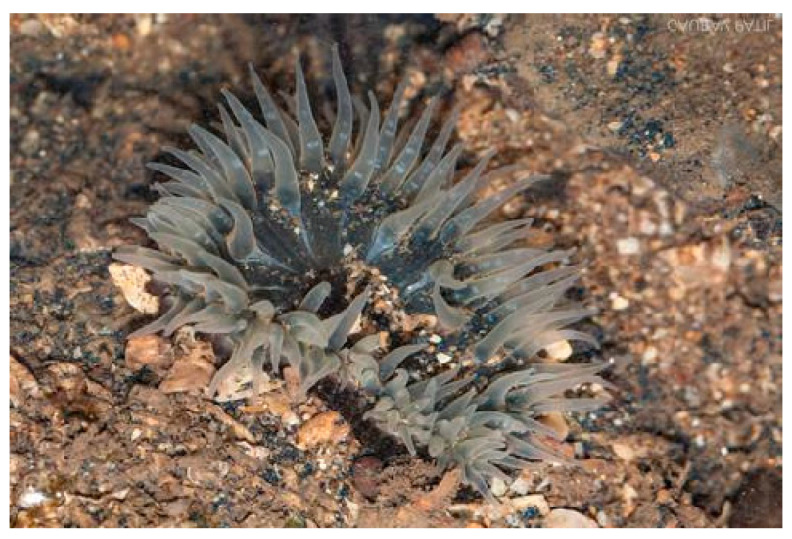
Specimen of *A. anjunae*. Author: Gaurav Patil (CC BY-NC). https://www.inaturalist.org/photos/61697779 (accessed on 20 September 2023).

*Anthopleura anjunae* (Den Hartog and Vennam, 1993; Cnidaria, Anthozoa, Actiniaria, Actiniidae; Figure 8) is a species distributed in India, China, the Indian Ocean, Japan, the North Pacific Ocean and South Korea. It is characterized anatomically by a disc of tentacles with a typical pale-yellow color, with darker yellow lines distributed along the length. A further feature concerns the presence of conspicuous button- to cup-shaped verrucae, which are stalked and normally adherent to foreign particles, such as fragments of shells, sand grains or barnacles [39].

Li et al. [40] and Wu et al. [41] examined the antitumor effect exerted by *A anjunae*’s AAP-H oligopeptide (YVPGP) in prostate cancer models in vitro and in vivo. In particular, it showed a dose- and time-dependent inhibitory rate on the viability of DU-145 cells, while having no effect on non-tumoral NIH-3T3 cells at the same concentration. This indicated that AAP-H was nontoxic and exhibited antitumor activity. Morphological analyses displayed drastic changes, such as cell shrinkage, nuclear DNA fragmentation and membrane blebbing. Flow cytometric and Western blot analyses showed that AAP-H impaired the cell cycle of DU-145 cells by blocking the progression of tumor cells from the S to the G_2_/M phase and was responsible for the dose-dependent down-regulation of p-AKT (Ser473), p-PI3K (p85) and p-mTOR (Ser2448), without altering the levels of total AKT and total PI3K. This was most likely linked to the induction of cell apoptosis, highlighted by the increased levels of the pro-apoptotic Bax factor, cytochrome c, initiator caspase 9 and executor caspase 3, and down-regulation of the anti-apoptotic Bcl-2 protein. Also, the cells’ mitochondrial transmembrane potential appeared dissipated. Confirmatory studies were also performed using nude mouse models, demonstrating the antitumor mechanism of APP-H on DU-145 xenografts and the involvement of the PI3K/AKT/mTOR signaling pathway in apoptosis promotion. In fact, the tumor growth rate of in the AAP-H-treated group was slower than the controls. In addition, in line with the in vitro results, immunohistochemistry showed the up-regulation of Bax and both initiator caspases 8 and 9 and executor caspases 3 and 7, and the concurrent down-regulation of the anti-apoptotic Bcl-xL protein.

### 4.3. Mollusca—Bivalvia

**Figure 9:**
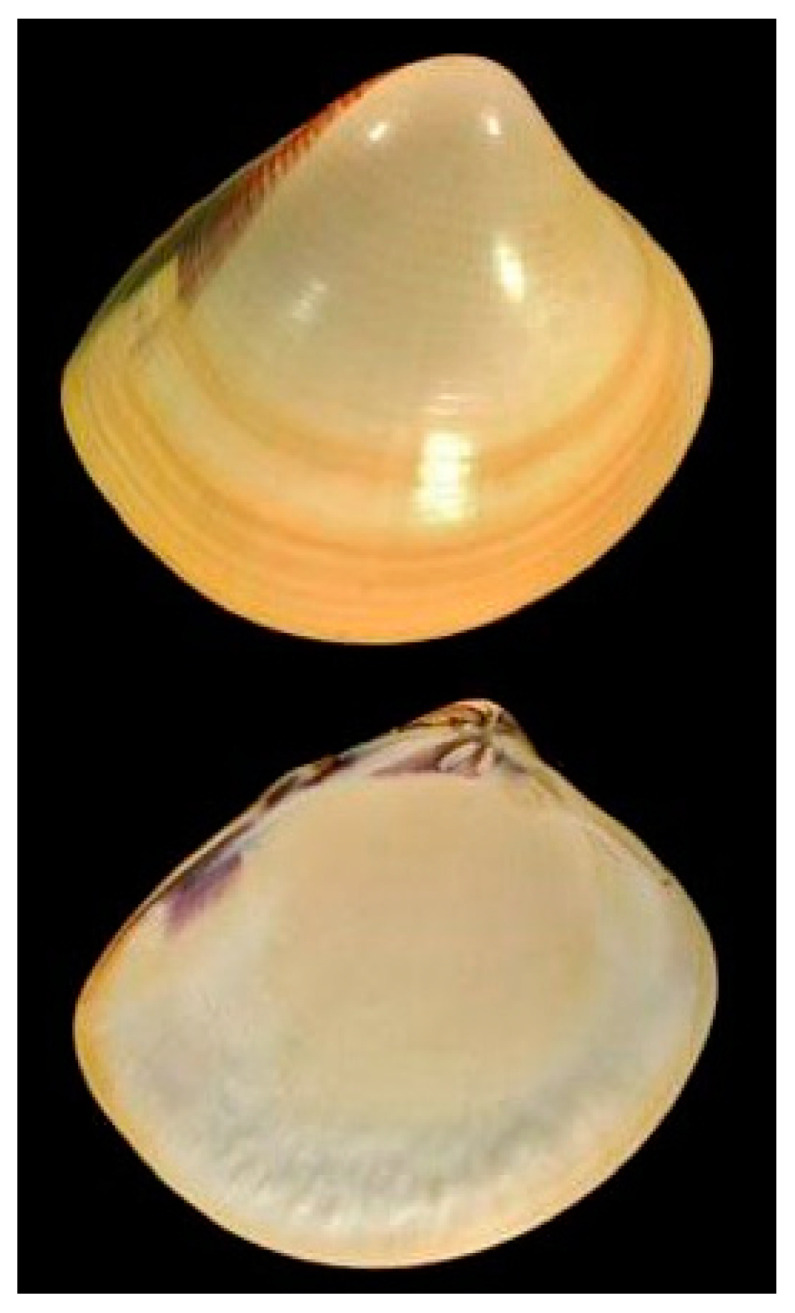
Specimen of *M. meretrix*. Author: Joop Trausel and Frans Slieker (CC BY-NC-SA 4.0). https://www.marinespecies.org/aphia.php?p=image&tid=224891&pic=67475 (accessed on 16 January 2023).

*Meretrix meretrix* (Linnaeus, 1758; Mollusca, Bivalvia, Venerida, Veneridae; Figure 9) is a benthic species distributed in the Indo-West Pacific region, from East Africa to the Philippines, and north to Japan and south to Indonesia. The morphology of this species is that of a strong, glossy, triangularly ovate and inflated shell, with round anterior and posterior edges. The outer shell is white, with a purplish tinge on the postero-dorsal slope. Typically found in the sand and mud of intertidal areas and adapted to less saline environments, it feeds on plankton and detritus [42].

In 2012, Wang et al. [43] purified from this organism a novel anticancer 15 kD polypeptide, Mere15, that exerted cytotoxicity against several human cancer cell lines derived from the human breast, cervix, colon, liver, pancreas and lung, the latter exhibiting the greatest effect. On the other hand, normal cells showed comparatively higher IC_50_ values. The mechanism of toxicity on A549 lung adenocarcinoma cells was associated with a G_2_/M phase arrest, followed by the promotion of the intrinsic apoptosis pathway, evidenced by membrane blebbing, loss of mitochondrial transmembrane potential, externalization of phosphatidylserine, chromosome condensation and DNA fragmentation, up-regulation of Bcl-2 and p53, and activation of caspase-3 and -9. Furthermore, Mere15 significantly suppressed the growth of human lung adenocarcinoma A549 xenograft in nude mice, with no apparent signs of toxicity. Subsequently, the analysis was extended by including in the study other lung cancer cell lines, that similar to A549 exhibited a significant growth decrease and apoptosis promotion, and demonstrated the additional anti-metastatic role played by Mere15 on these cells through the inhibition of adhesion, motility and invasion, mediated by the down-regulation of metalloproteinase-2 and -9 and Snail and the up-regulation of E-cadherin. An in vivo study also revealed that Mere15 inhibited tumor growth of NCI-H460 lung cancer cell xenografts in nude mice [44,45].

Another peptide extracted from *M. meretrix*, i.e., MM15 [46] (RKLAITEVDLERSETRLEAAEAKITELSEELAVVGNNCKALQNAVDQASQREDSYEETIRDLTQRLKDAENRAAEAERVVNKLQKEVDRLEDELLAEKEKYKQISDELDQTFAEFAGY), and exhibiting strong sequence homology with tropomyosin from several marine invertebrate species, was proven to have cytotoxic effects on different types of human tumor cells. Treatment of U87 glioma cells with recombinant MM15 determined cell cycle arrest at the G_2_/M phase, and programmed cell death by triggering tubulin polymerization and inducing the down-regulation of Bcl-2 and Bcl-xL and the increase in cleaved caspase-3 and cleaved PARP. Moreover, MM15 exerted a significant inhibitory effect on the motile and invasive behavior of U87 cells through down-regulation of the FAK/Akt/MMPs signalization. It is noteworthy that the peptide also significantly inhibited U87 cell proliferation and metastasis in vivo with little toxicity to the mice, thereby representing a promising anticancer candidate for the treatment of human glioblastoma.

**Figure 10:**
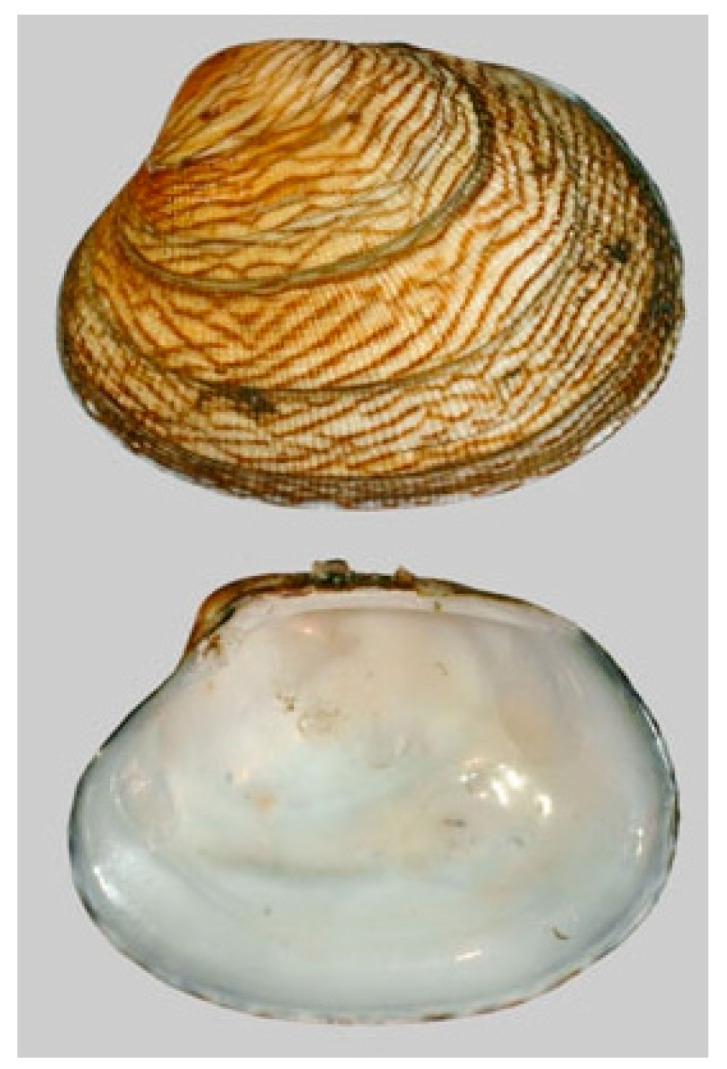
Specimen of *R. philippinarum*. Author: Joop Trausel and Frans Slieker (CC BY-NC-SA 4.0). https://www.marinespecies.org/photogallery.php?album=700&pic=106682 (accessed on 14 March 2023).

*Ruditapes philippinarum* (A. Adams and Reeve, 1850; Mollusca, Bivalvia, Venerida, Veneridae; Figure 10), also known as the Philippine clam or false clam, is native to the Indian and the Pacific Oceans, although for commercial reasons it has also been introduced in other sites in the Mediterranean Sea. Its life habitat is typically on shallow muddy bottoms of the sea. Morphologically speaking, the shell is brown, marbled or banded, and is characterized by coarse spiral ribs and radial furrows. The inside of the valves is often purplish or brownish.

Kim et al. [47] isolated the anticancer peptide AVLVDKQCPD from a fractionation of the α-chymotrypsin hydrolysates from *R. philippinarum*. It was found to induce cytotoxicity through apoptosis promotion, evaluated through the quantitation of the sub-G_0_G_1_ cell population on PC-3 prostate, A549 lung and MDA-MB231 breast tumor cells, but not on normal liver cancer cells. This peptide possesses six kinds of hydrophobic residues at its N-terminal. In light of the known crucial role against cancer cells played by the modulation of peptides’ hydrophobicity [48], some modifications of the sequence were supposed to improve its activity.

**Figure 11:**
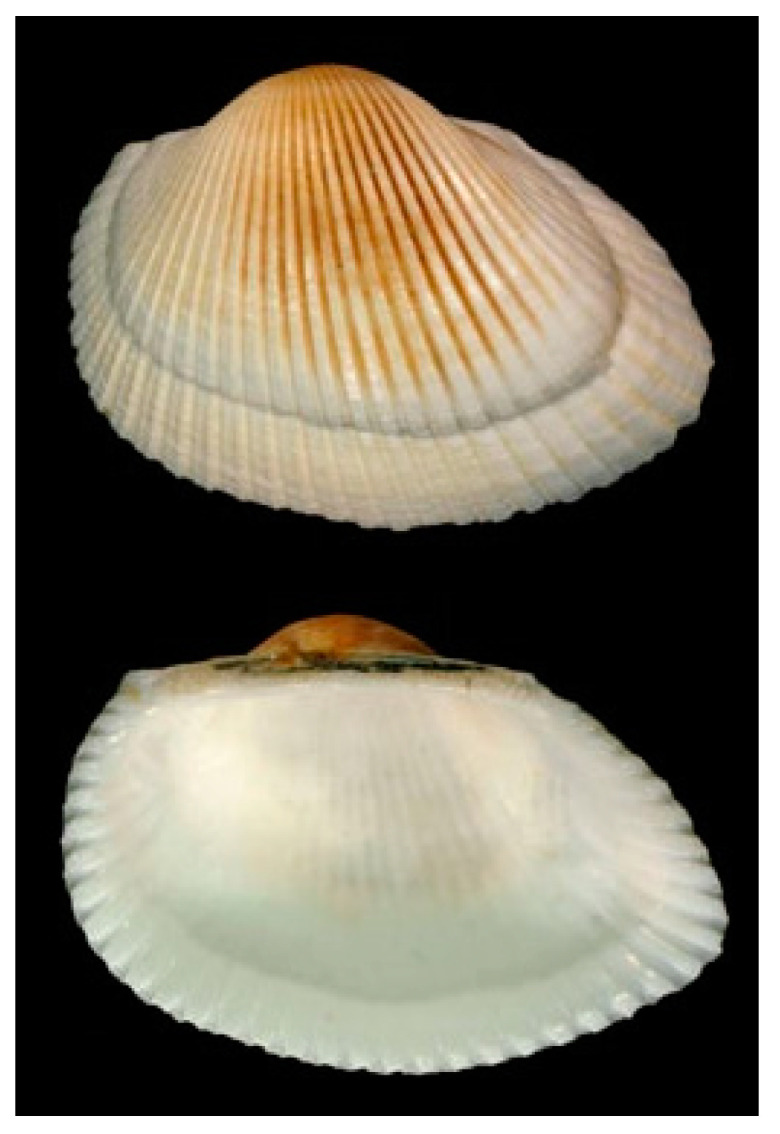
Specimen of *A. broughtonii*. Author: Joop Trausel and Frans Slieker (CC BY-NC-SA 4.0). https://www.marinespecies.org/aphia.php?p=image&tid=504357&pic=50786 (accessed on 6 March 2023).

*Arca inflata* (Reeve, 1844), renamed *Anadara broughtonii* (Schrenck, 1867; Mollusca, Bivalvia, Arcida, Arcidae; Figure 11), is a benthic and subtropical species, typically prevailing in Japan, the Gulf of Tartary and the Philippines. It displays a yellowish color, with brown and whitish shades, and a rounded shape.

In the hemolymph from this organism, Li et al. [49] identified a novel antitumor peptide, named P6 (WYIRKIRRFFKWLKKKLKKK, M.W. 2794.8 Da), rich in lysine residues and non-homologous to any previously discovered animal-derived anticancer peptide. P6 markedly inhibited growth and colony formation of HT-29, HCT116, SW620 and DLD-1 colon cancer cells in a concentration-dependent manner. The most sensitive cell lines being DLD-1 and HT-29, they were assayed to elucidate whether P6 induced apoptosis. The results obtained demonstrated that apoptosis was promoted through the activation of the p38-MAPK signaling pathway, and cell cycle arrest at the S/G2 transition occurred in both cell lines. In particular, P6 was proven to induce marked changes in the mitochondrial membrane potential and to up-regulate the intracellular Ca^++^ and reactive oxygen species (ROS) concentrations contextually increasing the expression levels of apoptosis-related proteins, including cleaved PARP and cleaved caspase-3 and the pro-apoptotic proteins Bak and cytochrome C. It is noteworthy that P6 also exhibited apoptosis-involved antitumor effects in a HT-29 tumor xenograft model with no toxic side effects on the mouse organs, thereby representing a promising molecule for therapeutic intervention against colorectal cancer.

**Figure 12:**
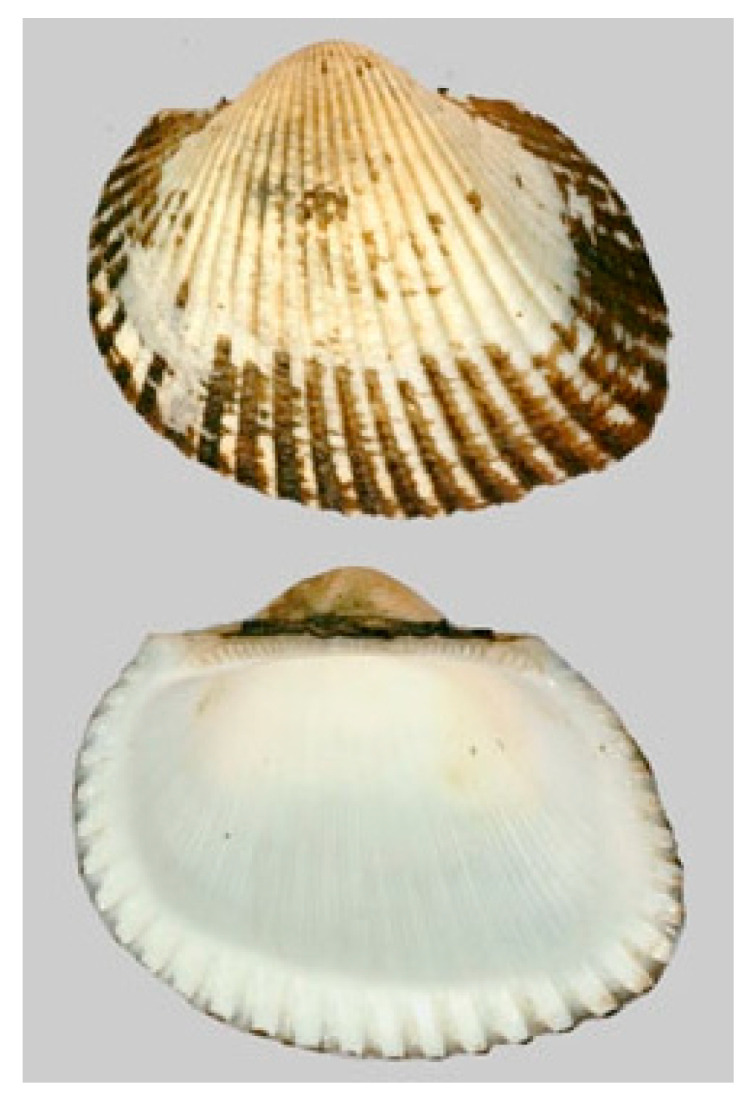
Specimen of *A. kagoshimensis*. Author: Joop Trausel and Frans Slieker (CC BY-NC-SA 4.0). https://www.marinespecies.org/photogallery.php?album=700&pic=50806 (accessed on 20 September 2023).

*Arca subcrenata* (Lischke, 1869), accepted as *Anadara kagoshimensis* (Tokunaga,1906; Mollusca, Bivalvia, Arcida, Arcidae; Figure 12), is typically found in shallow waters in the temperate zones of the western Pacific Ocean, but has also been detected in the Mediterranean Sea, Marmara Sea, Black Sea and Azov Sea. Being used for human consumption, for this reason it is widely farmed in China, Japan and Korea. Its morphology is very similar to that of a clam and is characterized by a thick, oval, white or creamy shell. It commonly lives buried in soft sediments, preferring mud or muddy sand, and shows good tolerance to low oxygen levels, variable temperatures and low salinity [50].

**Figure 13:**
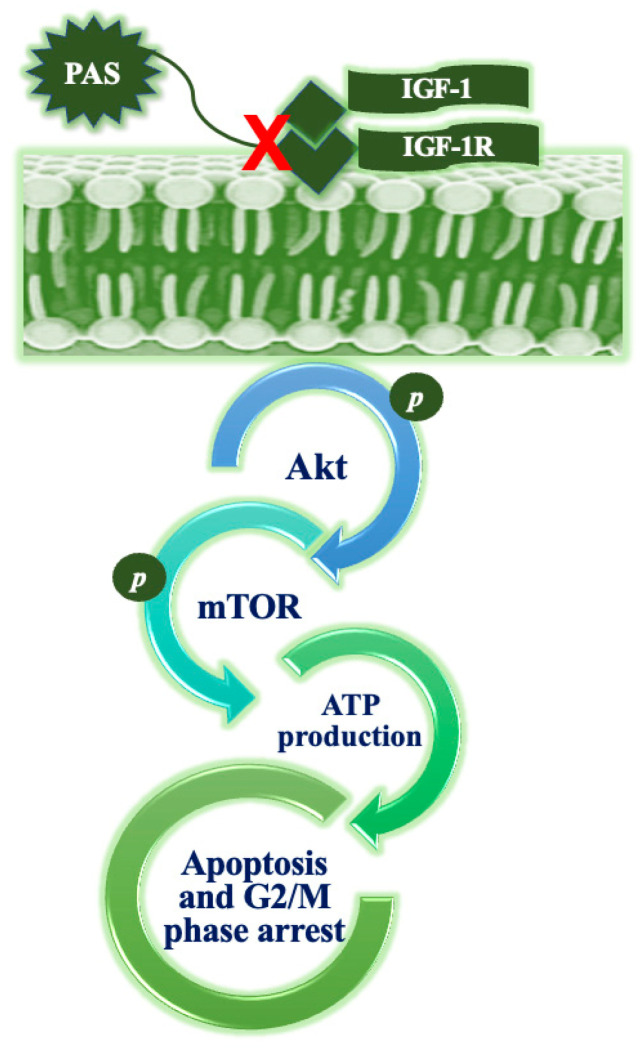
Scheme depicting the proposed mechanism of action of PAS, which induces cell cycle arrest and apoptosis downstream to the suppression of IGF-1R/Akt/mTOR signaling and ATP production. Redrawn from [52].

P2 (a.k.a. PAS) is a marine polypeptide fraction purified from *A. subcrenata*’s crude peptide extracts using DEAE Sepharose Fast Flow ion-exchange chromatography, mainly composed of arginine kinase, cartilage matrix protein-like isoform X1, retinal dehydrogenase 1-like isoform X2 and sarcoplasmic calcium-binding protein-like isoform X1. It was proven to inhibit the growth of several cancer cell lines in vitro and in vivo, with little cytotoxicity on normal cells and tissues. The investigation on HT-29 cells demonstrated that PAS significantly induced G2/M phase arrest and apoptosis involving the down-regulation of cyclin B1, cdc2, Bcl-2 and Ki 67, and the up-regulation of cleaved caspase 3, cleaved PARP and Bax. Apoptosis appeared to be dependent upon the marked depletion of ATP synthesis downstream for the inhibition of the IGF-1R/Akt/mTOR signalization (Figure 13) [51,52]. Interestingly, P2 was also found to be able to suppress the production of nitric oxide in LPS-induced RAW264.7 macrophages, as well as the secretion of the inflammatory cytokines IL-6 and TNF-α by HeLa cells. Due to its action on the down-regulation of the genes coding for such cytokines and on the inhibition of the COX-2 and iNOS-related pathways in HeLa cells, it is conceivable that P2 might interfere with tumor development by inhibiting the interplay between the tumor microenvironment and the pro-inflammatory mediators [53].

**Figure 14:**
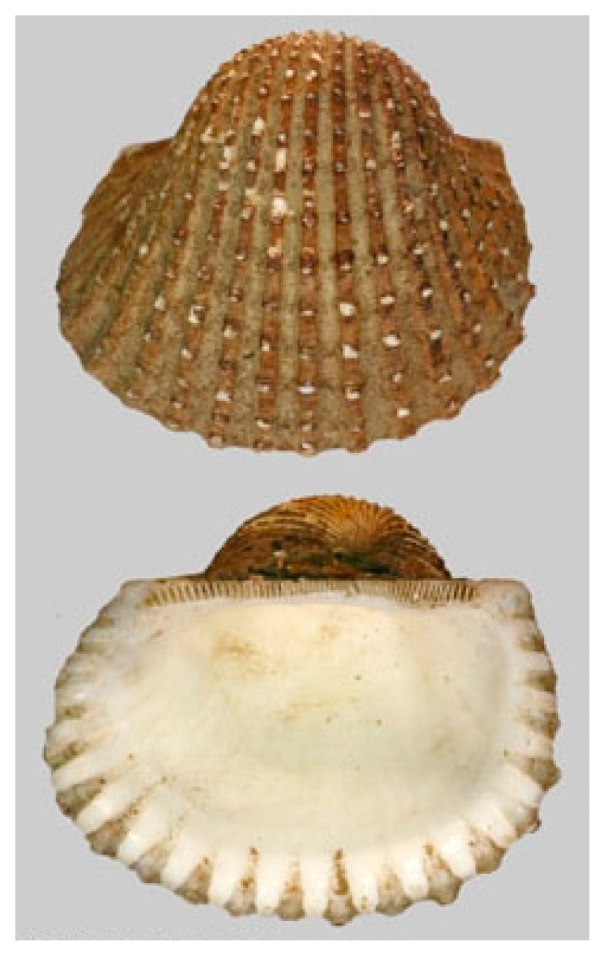
Specimen of *T. granosa*. Author: Joop Trausel and Frans Slieker (CC BY-NC-SA 4.0). https://www.marinespecies.org/photogallery.php?album=700&pic=51141 (accessed on 16 January 2023).

*Tegillarca granosa* (Linnaeus, 1758; Mollusca, Bivalvia, Arcida, Arcidae; Figure 14) is also known as blood heart or the blood mollusk, due to the typical presence of red hemoglobin liquid inside the tissues. It is distributed in the Indo-Pacific region from the east coast of South Africa to Southeast Asia, Australia, Polynesia and northern Japan. This species of benthic and brackish mollusks, which is typically used in aquaculture, lives buried in the sand at a water depth of one to two meters. Its shell, whose external color is white and yellowish-brown under the periostracum, is thick and solid with an ovate and swollen shape.

From the protein hydrolysate from *T. granosa*, Chi et al. [54] isolated the hydrophobic BCP-A peptide (WPP, M.W. 398.44) endowed with significant lipid peroxidation inhibitory and radical scavenging activity. In addition, BCP-A showed strong dose-dependent cytotoxicity against the HeLa, DU-145, H-1299 and, to a greater extent, PC-3 cancer cell lines. Further studies on the morphological features of acridine orange/ethidium bromide-stained PC-3 cells and on their externalization of phosphatidylserine, indicated the occurrence of apoptotic induction following their exposure to the peptide. Thus, the anti-tumoral mechanism of action of BCP-A may potentially be dual, that is, based upon a direct death-triggering effect on neoplastic cells and, also, indirectly due to the scavenging activity, which eliminating excessive intracellular ROS, prevents their cancer-promoting effect.

### 4.4. Mollusca—Gastropoda

**Figure 15:**
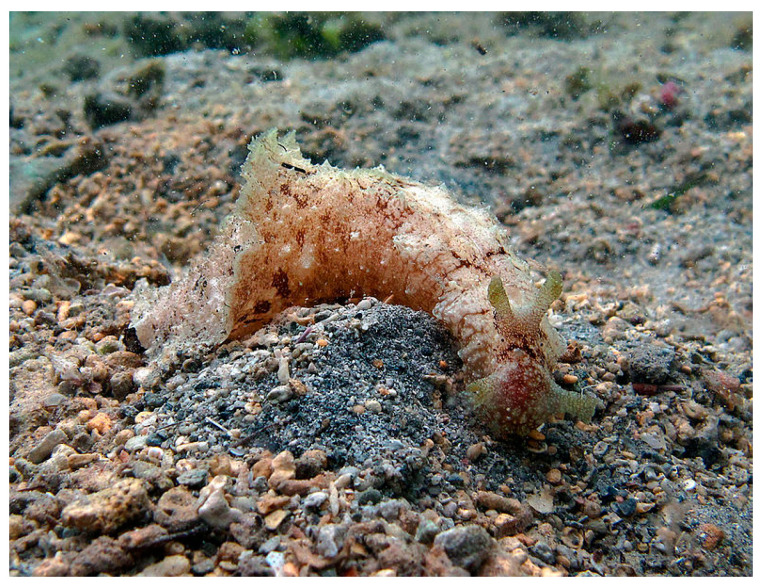
Specimen of *D. auricularia*. Author: Philippe Bourjon (CC BY-NC-SA 3.0). https://commons.wikimedia.org/wiki/File:Dolabella_auricularia.jpg (accessed on 16 January 2023).

*Dolabella auricularia* (Lightfoot, 1786; Mollusca, Gastropoda, Aplysiida, Aplysiidae) is a benthic species typical of the Indo Pacific area (Figure 15). These mollusks are commonly found among seaweeds and grass flats in shallow water environments, especially in sheltered bays and lagoons. The individuals in this species are herbivores, nocturnal feeders and simultaneous hermaphrodites, and their life span is about 16 months [55].

Dolastatins are natural peptides derived from *D. auricularia*, whose activity as antineoplastic agents has been the subject of numerous reports. Among them, the most studied members of the group are dolastatin (Dol)-10, consisting of four amino acids, namely dolavaline, valine, dolaisoleucine and dolaproine, and -15, consisting of seven amino acids, namely dolavaline, valine, N-methylvaline, proline, proline, 2-hydroxyisovaleric acid and dolapyrrolidone. A number of reports in the literature have demonstrated that Dol-10 was a strong inhibitor of the G_2_/M checkpoint and tubulin polymerization, and an apoptosis promoter in lymphoma, lung and prostate cancer cells, as evidenced by different endpoints, such as the generation of apoptotic bodies, the positivity in the TUNEL assay, the down-regulation of Bcl-2 and the up-regulation of p53. Of note, Dol-10 inhibited the growth of metastatic tumors from DU-145 prostate cancer cell xenografts in athymic mice [56,57,58,59,60]. Also, Dol-15 was proven to have apoptotic-promoting activity on myeloma and lung cancer cells, although less potent than Dol-10 in the latter case [59]. In addition, Sato et al. [61] showed that Dol-15 was able to induce cell cycle arrest at the G_2_/M checkpoint and stimulate apoptosis in myeloma cells through the activation of chk2 kinase and the concurrent inhibition of cdc25C phosphatase, which blocks the subsequent activation of cyclin B1/cdc2 activity required for the G_2_/M transition. The Dol-15 apoptotic effect on myeloma cells was also found to be associated with the mitochondrial- and Fas (CD95)-mediated pathway. The anti-mitotic mechanism of action of Dol-15 was examined in HeLa cells and the results obtained suggested that it induced a loss of tension across the kinetochore pairs, with consequent accumulation of the tension-sensing checkpoint protein BuBR1 at the kinetochores, thereby keeping the cells arrested in mitosis [62].

**Figure 16:**
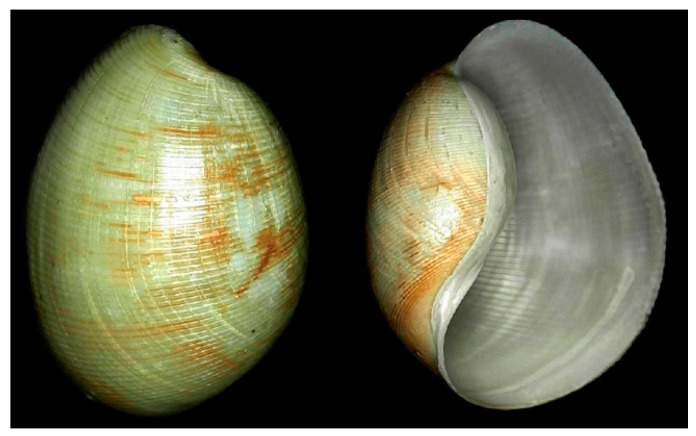
Specimen of *B. exarata*. Author: Kirsten Van Laethem (CC BY-NC-SA). https://www.marinespecies.org/aphia.php?p=image&tid=533828&pic=126086 (accessed on 6 March 2023).

*Bullacta exarata* (Philippi, 1849; Mollusca, Gastropoda, Cephalaspidea, Haminoeidae; Figure 16), to date accepted as *Bullacta caurina* (Benson, 1842), is a demersal, subtropical hermaphroditic species distributed in the Western Pacific area, in particular, China and Korea, whose habitat includes intertidal flats. Its common name is the Korean mud snail, and it is a commercially important species used as food in eastern China. It is endowed with a thick, white, spirally streaked shell, displaying a well-developed periostracum and a smooth and simple columella [63].

Specimens of the mollusk were submitted to trypsin digestion, ultrafiltration and Sephadex gel chromatography and, among the peaks of the hydrolysates obtained, the BEPT II fraction and the purified BEPT II-1 peptides (RAALAVVLGRGGPR and RDGDSCRGGGPV) exhibited anti-prostate cancer effects in vitro. This was evidenced by both the dose- and time-dependent inhibition of PC-3 cell proliferation and the morphological and Annexin V/propidium iodide (PI)-staining studies on the exposed PC-3 cells showing apoptosis-related changes, such as cell volume decrease and shrinkage, chromatin decondensation, the appearance of cytoplasmic blebs and the increase in the Annexin V-positive and PI-negative cell subpopulation [64].

### 4.5. Mollusca—Cephalopoda

**Figure 17:**
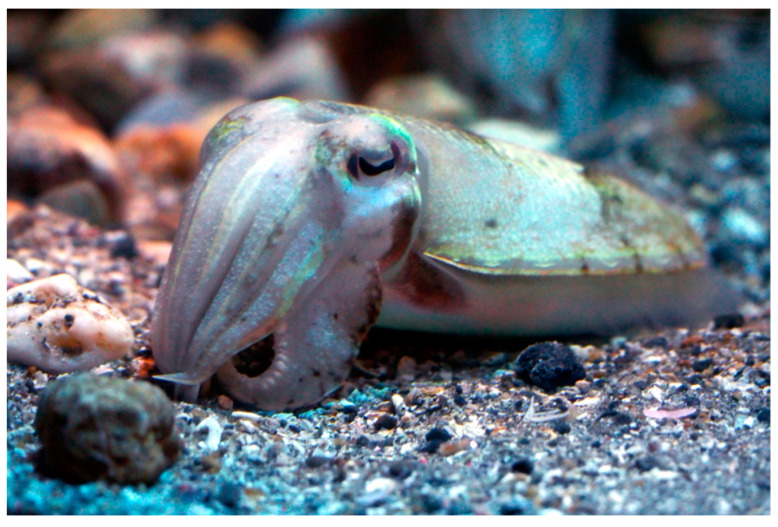
Specimen of *S. esculenta*. Author: Harum Koh (CC BY-SA 2.0). https://upload.wikimedia.org/wikipedia/commons/b/b1/Japan_squid%2C_Sepia_esculenta_%2815601195858%29.jpg (accessed on 16 January 2023).

*Sepia esculenta* (Hoyle,1885; Mollusca, Cephalopoda, Sepiida, Sepiidae; Figure 17), a.k.a. golden cuttlefish, is a subtropical, oceanodromous species, whose length can reach 18 cm, distributed in the North and Western Pacific Ocean in a depth range of 10–150 m, meaning that it is a nektobenthic organism [65].

Similar to BEPT II-1 from *B. exarata*, Huang et al. [66] isolated the peptide SHP (LKEENRRRRD) from a fraction of Sephadex gel filtered pepsin hydrolysate from *S. esculenta*’s ink, capable of exerting a significant time- and dose-dependent inhibitory effect on the proliferation of PC-3 prostate cancer cells and inducing apoptosis, as revealed by acridine orange/ethidium bromide and Annexin V/PI staining assays. Further investigation on the mechanism of SHP-dependent apoptotic promotion showed the involvement of the up-regulation of the Bax/Bcl2 ratio and p53 expression and an increase in the caspase-3 protein amount.

### 4.6. Anellida—Polychaeta

**Figure 18:**
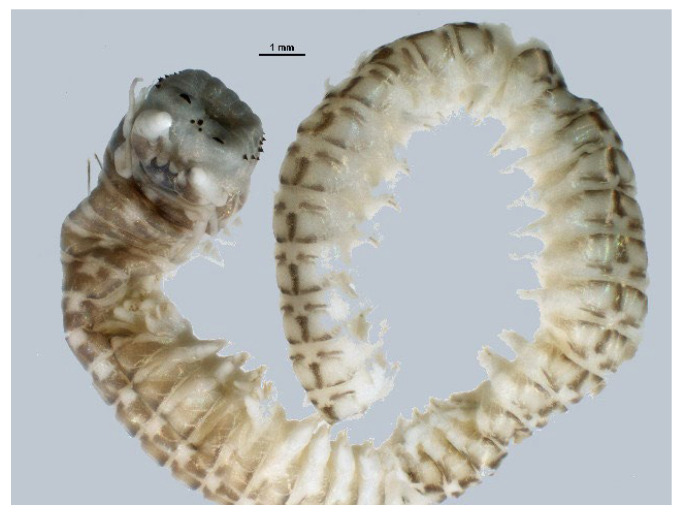
Specimen of *P. aibuhitensis*. Author: Hong Zhou (CC BY-NC SA 3.0). https://eol.org/media/30500878 (accessed on 6 March 2023).

*Perinereis aibuhitensis* (Grube, 1878; Annelida, Polychaeta, Phyllodocida, Nereididae; Figure 18) is a segmented ragworm living in sediments in the brackish and salty areas of China, Korea and the Philippines. It is characterized by an elongated, semi-cylindrical and ringed trunk. It is endowed with four eyes and powerful pincer jaws typical of the Nereididae family, and its color varies from brownish green to reddish brown [67].

The anticancer effect of PAP, the IEPGTVGMMF decapeptide from *P. aibuhitensis*, on H1299 human lung tumor cells was investigated by Jiang et al. [68]. PAP inhibited the proliferation of H1299 cells in a time- and dose-dependent manner through G_2_/M phase arrest, whereas it exerted no cytotoxic effects on NIH-3T3 fibroblasts. PAP also showed pro-apoptotic activity against H1299 cells, as demonstrated by acridine orange/ethidium bromide and Annexin V-PI staining assays and the up-regulation of the Bax/Bcl-2 ratio and caspase-3 and -9. Moreover, the expression levels of Nm23-H1 nucleoside diphosphate kinase and VEGF decreased significantly at the increase of the PAP concentration, indicating that the peptide might inhibit tumor growth and angiogenesis.

### 4.7. Arthropoda—Malacostraca

**Figure 19:**
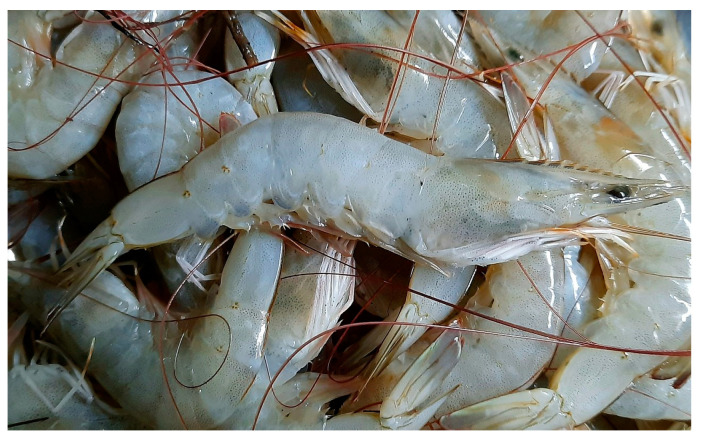
Specimens of *L. vannamei*. Author: Fotokannan (CC BY-SA 4.0). https://upload.wikimedia.org/wikipedia/commons/8/85/Pacific_white_shrimp.jpg?uselang=it (accessed on 20 September 2023).

*Litopenaeus vannamei* (Boone, 1931; Arthropoda, Malacostraca, Decapoda, Penaeidea; Figure 19), whose accepted name is *Penaeus vannamei*, is also known as the tropical shrimp or Pacific white shrimp, and plays an important role in human nutrition and the fish trade, being widely used in the aquaculture context. It is distributed along the coasts of the Pacific Ocean, from California to Peru, and normally inhabits waters whose temperature does not drop below 20 °C down to a depth of 72 m. Its color is translucent, bluish or olive, with dark, reddish-brown bands on the antennula and white legs [69].

A number of hemocyanin-derived peptides from this species have shown antibacterial properties; among them, two peptides have been proven to possess anticancer potential. The hydrophobic cationic peptide B11 (RIRDAIAHGYIVDKV) significantly inhibited the proliferation of human cervical HeLa, hepatocellular carcinoma HepG2 and esophageal cancer EC109 cell lines, but not normal liver cell lines, by inducing apoptosis as demonstrated by morphological observations and Annexin V-PI staining assays. Moreover, this peptide was imported into the mitochondria of HeLa cells, thereby causing mitochondrial dysfunction, i.e., the loss of transmembrane potential. At the protein expression level, there was an increase in the caspase-3 and -9 and the pro-apoptotic Bax factor; however, a decrease was observed in the expression level of the pro-survival Bcl-2 factor [70]. Similar results were obtained with the cationic peptide LvHemB1 (DVNFLLHKIYGNIRY), which was also found to interact with the mitochondrial voltage-dependent anion channel 1, a mitochondrial gatekeeper that controls energy metabolism and apoptosis [71] and induces ROS up-regulation [72].

**Figure 20:**
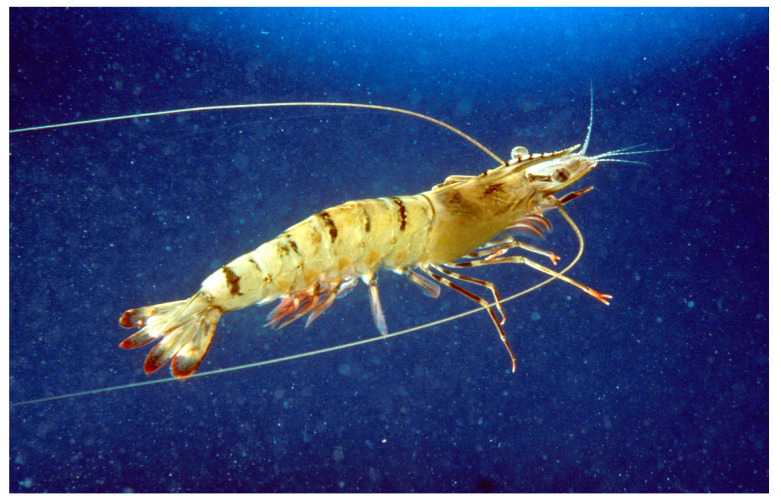
Specimen of *P. monodon*. Author: CSIRO (CC-BY-3.0). https://upload.wikimedia.org/wikipedia/commons/2/2e/CSIRO_ScienceImage_2992_The_Giant_Tiger_Prawn.jpg (accessed on 14 March 2023).

*Penaeus monodon* (Fabricius, 1798; Arthropoda, Malacostraca, Decapoda, Penaeidae; Figure 20), a.k.a. the Indo-Pacific Giant Shrimp, is an edible species mostly distributed in the tropical waters on the muddy bottoms of the Pacific and Indian Oceans. It is characterized by large dimensions and a dark color, which can vary according to the living conditions, with light, yellow and black streaks and brown or gray antennae [73].

The shrimp-derived anti-lipopolysaccharide factor (SALF; ECKFTVKPYLKRFQVYYKGRMWCPNH_2_), an antimicrobial peptide, was administered to HeLa tumor cells and found to be able to inhibit their proliferation and reduce colony formation in a soft agar assay. Interestingly, an enhanced effect was observed when SALF and cisplatin were used in combination, and such effect was correlated to the angiogenic and metastatic activities of HeLa cells, whose growth in soft agar was drastically reduced upon treatment. Exposure to SALF also caused the alteration and rupture of the cell membrane, as shown by the TEM analyses, and the death receptor/NF-κB signaling pathway-linked promotion of apoptosis with the time-dependent activation of caspases 6, 7 and 9 and the down-regulation of Bcl-2 and NF-κB. Ultimately, SALF exerted a significant tumor-suppressive effect in mice with HeLa-derived tumor xenografts in an in vivo analysis, thereby representing a promising candidate for cervical cancer treatment [74].

**Figure 21:**
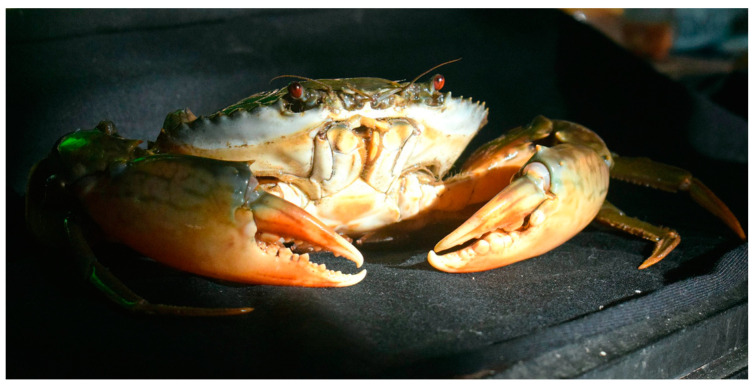
Specimen of *S. paramamosain*. Author: Wibowo Djatmiko (CC BY-SA 4.0). https://en.wikipedia.org/wiki/Scylla_paramamosain#/media/File:Scyl_param_180225-5311834_mrd.JPG (accessed on 6 March 2023).

*Scylla paramamosain* (Estampador, 1950; Arthropoda, Malacostraca, Decapoda, Portunidae; Figure 21) is a benthic, tropical species, typically distributed in the South China Sea and commonly farmed in aquaculture, characterized by a green to greenish, blue-colored carapace, with a usually pale yellow or yellowish-orange undersurface [75].

It is known that rScyreprocin (MKEDSNILDKTAKMTKQNKALLFTAGGAAAFMAGYYYYHCNYRNPAPKKSGSTTSQDKTDAQAVQSIPSPSGNKGKESKDPKVK), a cationic peptide identified in this species, exhibits anti-microbial and anti-fungal activities [76]. Further investigation showed that this peptide inhibited the growth, migration and colony formation ability of different cancer cell lines, i.e., lung cancer H460, liver cancer HepG2, cervical cancer HeLa, bladder cancer T24 and prostate cancer Du145 cells, whilst being non-toxic to cell lines of normal origin, such as HFL1 lung fibroblasts and L02 liver cells. Moreover, rScyreprocin was proven to be a cell-penetrating peptide due to its ability to disrupt plasma membranes and, once internalized, to promote apoptosis through ROS up-regulation leading to endoplasmic reticulum stress and Ca^++^ release, which further caused mitochondria dysfunction, loss of mitochondrial transmembrane potential (MMP) and activation of the caspase-3 cascade. Also, the peptide exerted a promising inhibitory effect on the growth of H460 xenografts in nude mice models, by inducing significant necrosis and apoptosis in the tissues and down regulating the proliferation of tumor cells [77].

**Figure 22:**
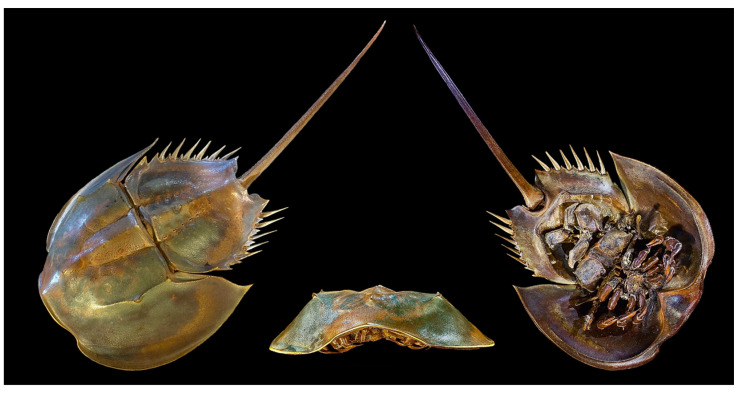
An example of the horseshoe crab, *Tachypleus tridentatus*. Author: Didier Descouens (CC BY-SA 4.0). https://upload.wikimedia.org/wikipedia/commons/f/f4/Limules.jpg?uselang=it (accessed on 14 March 2023).

Horseshoe crabs (Figure 22) are marine arthropods belonging to the order Xiphosura and the family Limulidae, used for nutritional purposes in Asia. Their habitat includes shallow coastal waters and, especially, muddy bottoms. Their body is protected by a hard carapace, and they possess small appendages, called chelicerae, for carrying food into their mouths and five successive pairs used for locomotion. The last pair of legs, in particular, is used for propelling the crab when walking on the ocean floor [78].

Tachyplesin (KWCFRVCYRGICYRRCR) is a cationic peptide obtained from the horseshoe crab, *Tachypleus tridentatus*, and endowed with anti-viral and anti-coagulative properties. The exposure of human hepatocarcinoma SMMC-7721 cells to tachyplesin was found to impair their mitotic index and revert their morphological and ultrastructural aspects to those of normal differentiated epithelial cells. In addition, at the biochemical and molecular levels, tachyplesin determined the increase in tyrosine aminotransferase activity and the decrease in that of γ-glutamyltransferase and, also, of the levels of α-fetoprotein and PCNA markers, while increasing the expression of p21^WAF1/CIP1^ and down regulating the c-myc protein, all being signs of malignant phenotype reversion and the induction of terminal differentiation [79]. Further cell cycle analysis of tachyplesin-treated SMMC-7721 cells revealed that the peptide can induce the differentiation-addressed G_0_/G_1_ arrest, also via the down-regulation of the levels of mutant p53, cyclin D1 and CDK4 proteins and the up-regulation of those of the p21 protein [80]. In vitro antitumor activity was also exerted by the peptide on human BGC-823 gastric cancer cells, where, in addition, the positive rate of c-erbB-2 expression decreased and the P16 protein level increased upon treatment [81]. When administered to glioma stem cells derived from the U251 cell line, tachyplesin induced the decrease in proliferation and impairment of the integrity of the plasmalemma by disrupting the lipid bilayer with its amphipathic helices [82]. Quantitative proteomic profiling demonstrated that in this glioma cell line the peptide altered the cellular metabolism mainly through the down-regulation of the major lysosomal hydrolases, such as cathepsin A, B and D, and the up-regulation of DNA topoisomerase 2α, thereby conceivably inhibiting cell migration and promoting apoptosis [83]. More recent results have enriched the list of tachyplesin-sensitive cells, demonstrating that the peptide also significantly inhibited proliferation and induced apoptosis in non-small cell lung cancer A549 and H460 cells, as confirmed by the increase in cleaved PARP and cleaved caspase-8 and the decrease in total BID and caspase-8. The molecular basis of apoptosis promotion in these cells was conceivably found in the increased expression of Fas and FasL, involved in the death receptor pathway, and the p-RIPK1 protein, involved in the necroptotic pathway, which might be responsible for cell membrane disruption and nucleus condensation. Evidence was also produced that the combination of tachyplesin and cisplatin significantly suppressed migration and improved the sensitivity to cisplatin in cisplatin-resistant A549/DDP cells [84].

### 4.8. Echinodermata

Sea cucumbers, which belong to the phylum Echinodermata and the class Holothuroidea, are marine organisms characterized by an elongated body and leathery skin, normally distributed on the seabed throughout the world. They are endowed with an endoskeleton of calcified structures reduced to microscopic ossicles, which can sometimes be enlarged into flattened plates. Ecologically, sea cucumbers are of particular importance for nutrient recycling and are used as nutritious and functional foods themselves [85]. From the freeze-dried intestine of Chinese sea cucumbers, Wei at al. [86] prepared a mixture called the sea cucumber intestinal peptide (SCIP), rich in hydrophobic and branched-chain amino acids, using alkaline enzymatic hydrolysis. SCIP was proven to inhibit, in a dose-dependent way, the growth of MCF-7 breast tumor cell xenografts in zebrafish. Moreover, flow cytometric and biochemical assays demonstrated that SCIP induced MCF-7 programmed cell death via the up-regulation of pro-apoptotic proteins, such as Bax, cleaved caspase-9 and -3 and cytochrome c (markers of the endogenous pathway) and the down-regulation of the anti-apoptotic Bcl-2 protein. In addition, promotion of cell apoptosis was found to be linked to SCIP-induced inhibition of the PI3K/AKT signaling pathway.

### 4.9. Chordata—Ascidiacea

**Figure 23:**
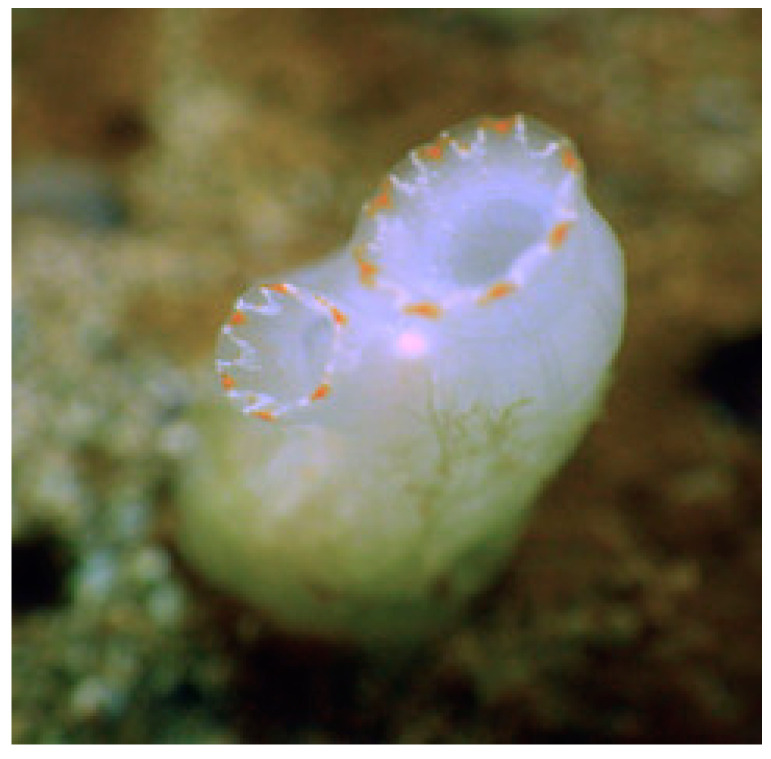
Specimen of *C. savignyi*. Author: Robin Gwen Agarwal (CC BY-NC). https://inaturalist.nz/photos/1083563 (accessed on 20 September 2023).

*Ciona savignyi* (Herdman, 1882; Chordata, Ascidiacea, Phlebobranchia, Cionidae; Figure 23) is a cosmopolitan, (e.g., found in Argentina, the North Pacific Ocean, the South Pacific Ocean, the South Atlantic Ocean, Spain, the Tasman Sea) filter feeder, sessile, solitary and tropical species. It is hermaphroditic, and both cross- and self-fertilization are typical [87].

From this organism, in 2012, Cheng et al. [88] purified CS5931, a novel polypeptide of about 6 kDa with the N-terminal partial sequence MVVCPDGQSECPDGN, able to exert a strong cytotoxic effect against several cancer cell lines, with HCT-8 colorectal carcinoma cells being the most sensitive to the treatment. Exposure of this cell line to CS5931 determined a G_2_/S phase arrest, with a decrease in the G_0_/G_1_ phase population. Further analyses demonstrated that the peptide damaged cell plasmalemma and induced dissipation of MMP, the release of cytochrome c, chromatin condensation and nuclear fragmentation, all markers of the onset of mitochondrial-mediated apoptosis, as also confirmed by the up-regulation of apoptotic proteins and the activation of caspase-3 and -9. Subsequently, CS5931 was proven to be anti-angiogenic, both in vitro and in vivo. In fact, it was able to inhibit the proliferation, migration and formation of capillary-like structures by human umbilical vein endothelial cells in a dose-dependent manner, also down regulating the expression of the vascular endothelial growth factor (VEGF) and the release of MMP-2 and -9. In addition, the peptide impaired the development of sub-intestinal vessels in zebrafish embryo [89].

### 4.10. Chordata—Elasmobranchii

**Figure 24:**
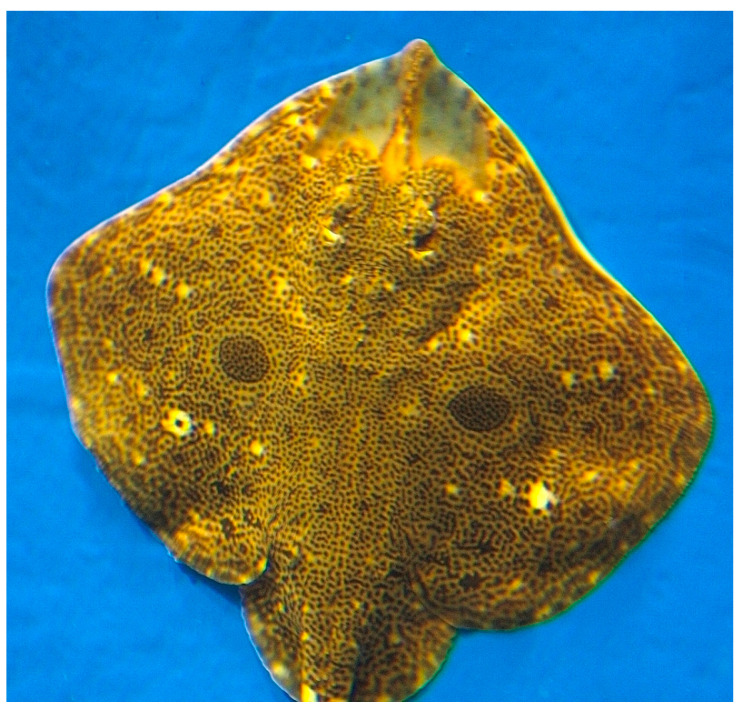
Specimen of *R. porosa*. (CC-BY-SA-2.5). https://en.wikipedia.org/wiki/Ocellate_spot_skate#/media/File:Okamejei_kenojei2.jpg (accessed on 14 March 2023).

*Raja porosa* (Günther, 1874; Chordata, Elasmobranchii, Rajiformes, Rajidae; Figure 24), to date accepted as *Okamejei kenojei* (Bürger in Müller and Henle, 1841) and also known as the ocellated skate, spiny rasp skate or dark skate, is a species commonly found in the northwestern Pacific Ocean. The shape and coloring of its body tends to be flat and with different shades of yellow and brown. It is a bottom-feeding carnivore that consumes shrimp, fish, crabs, small amounts of amphipods, mysids, cephalopods, euphausids, copepods, isopods and polychaetes [90].

In 2016, Pan et al. [91] isolated the hydrophobic residues-rich hexapeptide FIMGPY from *R. porosa*’s cartilage protein hydrolysate via ultrafiltration and chromatography and evaluated its anti-proliferative activity on HeLa tumor cells, which was found to be dose-dependent with an IC_50_ of 4.81 mg/mL. Acridine orange/ethidium bromide fluorescence staining, DNA fragmentation and flow cytometry assays demonstrated that the inhibitory effect of the peptide was based upon apoptosis induction, as also confirmed by the up-regulation of the Bax/Bcl-2 ratio and the activation of caspase-3.

### 4.11. Chordata—Teleostei

**Figure 25:**
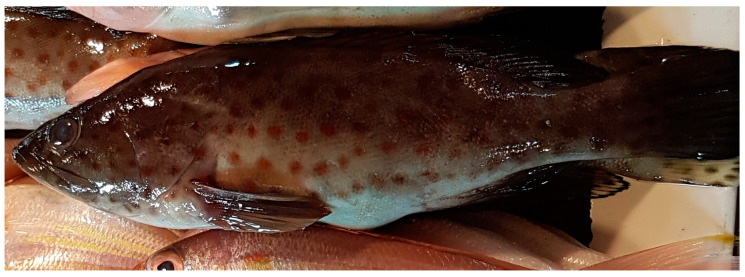
Specimen of *E. coioides* (CC-BY-SA-4.0). https://commons.wikimedia.org/wiki/File:Epinephelus_coioides_%28Orange_spotted_grouper%29_in_the_Philippines.jpg (accessed on 20 September 2023).

*Epinephelus coioides* (Hamilton, 1822; Chordata, Teleostei, Perciformes, Serranidae; Figure 25), also named the orange-spotted grouper, is a species of bony fish of economic importance, distributed from the Mediterranean Sea to the Indian ocean. This species is a protogynous hermaphrodite, although some authors have reported it to be a diandric protogynous teleost, with two types of males: primary and secondary. The first one develops directly from juveniles, while the secondary one develops after a sex change in female fish [92,93]. Usually, each individual is up to 1 m long, and characterized by dark vertical bands, with a beige or light brown livery and reddish brown or orange dots distributed completely over the whole body. Normally they are solitary animals who feed on small fish or shrimp and/or crabs and prefer reef areas with murky and brackish water, whereas the juvenile individuals inhabit shallow waters.

Epinecidin-1 (Epi-1) is a cationic 21-aminoacid antimicrobial peptide (GFIFHIIKGLFHAGKMIHGLV) identified from the grouper *E. coioides*, capable of interacting with the anionic phospholipids present in the plasmalemma of bacterial cells, thus impairing its structure and function. Lin et al. [94] demonstrated that the peptide was able to inhibit the viability and clonal growth of different cancer cell lines, also in this case inducing cell lysis due to the likely formation of membrane pores, as revealed by observations using a scanning electron microscope. In addition, the cell membrane lytic effect in case of exposure of HT1080 fibrosarcoma cells to Epi-1 appeared to trigger anti-necrosis via down-regulation of the necrosis-related genes. Concerning Epi-1-mediated inhibition of the proliferation of human leukemia U937 cells, the data produced by Chen et al. [95] revealed a correlation between the treatment and the mitochondrial dysfunction based upon the increase in the ADP/ATP ratio. Moreover, Epi-1 was found to induce apoptosis linked to caspases-3, -8 and -9 activation, as also supported by the DNA fragmentation assay and flow cytometric analysis of annexin V-FITC/PI staining. Real-time RT-PCR data showed the up-regulation of interleukin-related genes, such as those coding for TNF-α, IL-10, interferon (INF)-r, p53, IL-15 and IL-6, thereby suggesting that Epi-1 may have pleiotropic effects, both pro-apoptotic and immunostimulatory, on selected cell lines. As a further confirmation of the heterogeneity of Epi-1 activities on different cancer cells, Su et al. [96,97] found that, when administered to U87MG glioblastoma or SW982 synovial sarcoma cells, the peptide promoted mitochondrial hyperpolarization and the subsequent production of DNA-damaging ROS, which, in turn, led to necrotic, and not apoptotic, cell death. Additionally, in the SW982 cell model, the Epi-1-mediated calcium overload was found to be responsible for the activation of cell necrosis-linked calpain, causing mitochondrial damage and the overproduction of ROS, determining the down-regulation of the antioxidant protein supply.

**Figure 26:**
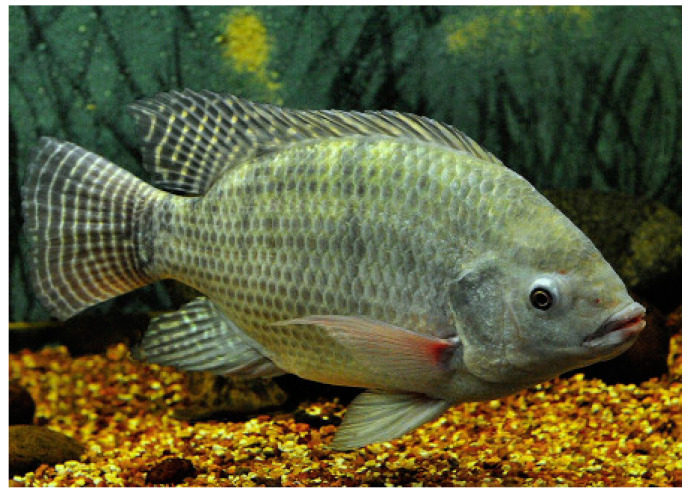
Specimen of *O. niloticus*. Author: Germano Roberto Schüür (CC-BY-SA-4.0). https://commons.wikimedia.org/w/index.php?curid=40488546 (accessed on 20 September 2023).

*Oreochromis niloticus* (Linnaeus, 1758; Chordata, Teleostei, Cichliformes, Cichlidae; Figure 26) is an euryhaline and gregarious species populating brackish waters, which are still or with small currents, but also in lakes, rivers, ponds, marshes and lagoons, in muddy and abundantly vegetated bottoms. Historically, it is a species of major interest for artisanal fishing in Africa, which is gaining increasing importance in aquaculture and aquaponics. Its body is typically disc shaped and laterally compressed with a large head, short snout and full lips. Like other cichlids, its lower pharyngeal bones are fused into a single tooth-bearing structure. The livery is gray with blue, green or yellowish-brown reflections, darker on the back and lighter on the sides towards the belly, which is white or yellowish white. On the flanks, dark vertical stripes are easy to spot. A distinctive feature is the opercular spot, which can be invisible depending on the emotional or physiological state of the individual. Typically, *O. niloticus* has a long dorsal fin, and a lateral line that often breaks towards the end of the dorsal fin and starts again two or three rows of scales below [98].

Ting et al. [99] showed that *O. niloticus*’s piscidin-4 peptide (TP4: FFRHLFRGAKAIFRGARQGXRAHKVVSRYRNRDVPETDNNQEEP [100]), endowed with a amphiphilic α-helical conformation structure and belonging to an antimicrobial peptide family first identified by Chinchar et al. [101] in the mast cells of the hybrid striped bass (*Morone saxatilis* × *Morone chrysops*), exerted a microtubule-destabilizing activity, which could be the mechanistic requirement for the TP4-mediated death of A549 lung carcinoma cells. Additional studies have demonstrated that the peptide was also active on triple-negative breast cancer cells, glioblastoma cells and SW982 human synovial sarcoma cells [102,103,104,105,106,107]. The data reported for synovial tumor cells indicated the promotion of necrosis via the induction of calcium overload, mitochondrial hyperpolarization, ROS accumulation and impairment of the antioxidant defense. In triple-negative breast and lung cancer cells, TP4 was proven to induce damage via the ERK/FOSB/cJUN axis controlled by Ca^2+^ signaling, leading to the selective binding of TP4 to the mitochondria; also, the activities of both JNKs and p38 MAPKs were inhibited. TP4 was found to integrate into mitochondria and directly interact with the adenine nucleotide translocator 2, which is essential for adenine nucleotide exchange across the inner membrane, thereby inducing the disruption of cellular energy metabolism. On the other hand, transcriptomic analysis revealed that FOSB activation disrupted cytoskeletal and membrane integrity and, in turn, promoted the expression of *PCDHB13*, coding for protocadherin-β13, which caused defects in microtubule assembly. Of note, up-regulation of FOSB and PCDHB13 diminished cell survival in vitro and in a zebrafish xenotransplantation model.

Limited studies on their anticancer role were also carried out on two other peptides in this family, i.e., piscidin-1 (TP1: FFHHIFRGIVHVGKTIHRLVTG) and piscidin-3 (TP3: FFHHIFRGIVHVGKTIHRLVTG). The first one, isolated from the mast cells of the hybrid striped bass (*Morone saxatilis* × *M. chrysops*), was proven to decrease viability and inhibit in vitro colony formation and motility of HT1080 fibrosarcoma cells, leading to the appearance of apoptotic phenomena. Apoptosis was also induced by TP1 on osteosarcoma cells, preceded by the up-regulation of mitochondrial ROS, the reduction in mitochondrial antioxidant manganese superoxide dismutase and MMP and the decrease in ATP production [108,109].

**Figure 27:**
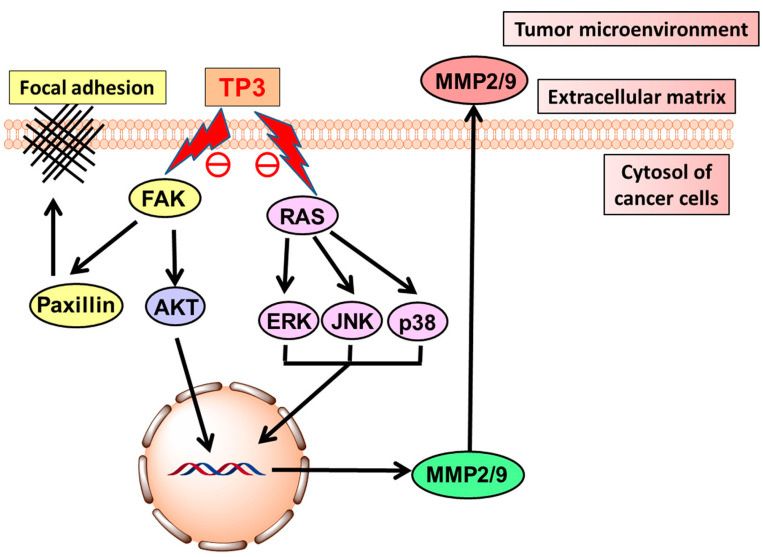
Proposed mechanism of action of TP3 on brain cancer cells, based upon the inhibition of FAK and RAS signalization, leading to the suppression of metalloproteases MMP2 and MMP9 in the tumor microenvironment [110] (CC-BY-4.0).

On the other hand, TP3 acted as an anti-adhesive and anti-invasive peptide on glioblastoma cell lines. The proposed mechanism of action for the inhibition of cell motility (Figure 27) hypothesizes that TP3 may suppress RAS activity and the downstream phosphorylation of ERK, p38 and JNK, and FAK activity, leading to the down-regulation of AKT. Both inhibitory events may determine the inhibition of the secretion of the metalloproteases MMP2 and MMP9, which, when present, cause the proteolysis of extracellular matrix components, and facilitate tumor cell invasion [110].

**Figure 28:**
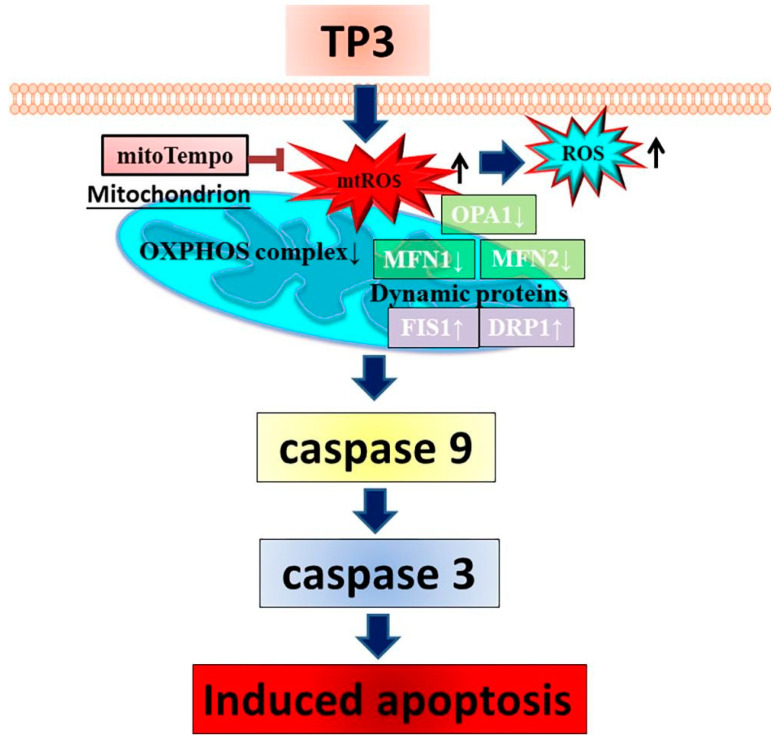
Proposed mechanism of action of TP3 on osteosarcoma cells, based upon the elevation of mitochondrial ROS production (↑), impairment of the activities of OXPHOS complexes and induction of caspase-9/3-mediated apoptosis. TP3-promoted apoptosis is also dependent upon the modulation of the expression levels of proteins associated with mitochondrial dynamics, such as OPA1, MFN1/2, FIS1 and DRP1. They lead to enhanced mitochondrial fission and, ultimately, to the destruction of mitochondrial function. The reducing agent mitoTempo may counteract ROS-mediated apoptosis [111].

Moreover, TP3 was also proven to reduce the viability of MG63 osteosarcoma cells, by inducing mitochondrial ROS overproduction, which led to disturbances in the MMP and to the activation of the caspase 9-mediated intrinsic apoptotic pathway (Figure 28) [111].

**Figure 29:**
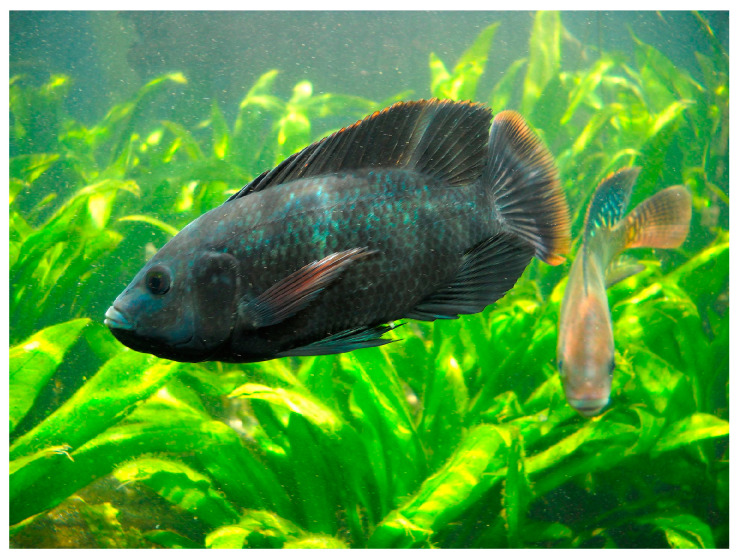
Specimen of *O. mossambicus* (CC-BY-SA-3.0). https://commons.wikimedia.org/wiki/File:Oreochromis_mossambicus.jpg (accessed on 6 March 2023).

*Oreochromis mossambicus* (Peters, 1852; Chordata, Teleostei, Cichliformes, Cichlidae; Figure 29), known as the Mozambique tilapia, is a bony fish native to south-eastern Africa and, subsequently, introduced to all continents (except Antarctica). This species is adaptable to various types of environments and normally lives in still waters or with very slow currents in muddy bottoms and, being euryhaline, it can also live in coastal ponds and in estuaries not communicating with the sea. It can survive and reproduce in marine salinity and has shown the ability to resist very low levels of dissolved oxygen and temperature ranges between 8 and 42 °C. Its body is laterally flattened and oval, the snout is elongated (pointed in adult males), the mouth is large with full lips. The color is typically darker silver, gray on the back and yellowish white on the belly, with blue, yellowish or greenish reflections. Furthermore, a particular feature is the presence of dark spots on the sides, a dark spot on the operculum and inclined light spots on the dorsal and anal fins [112].

The isoform hepcidin TH2-3 (QSHLSLCRWCCNCCRSNKGC), an antimicrobial peptide isolated from *O. mossambicus*, was found to selectively inhibit human HT1080 fibrosarcoma cells’ proliferation and migration. Of note, the peptide induced cell lysis by targeted membrane disruption through hole forming and, moreover, induced the down-regulation of *JUN,* which is conceivably involved in the apoptotic death of cancer cells [113].

**Figure 30:**
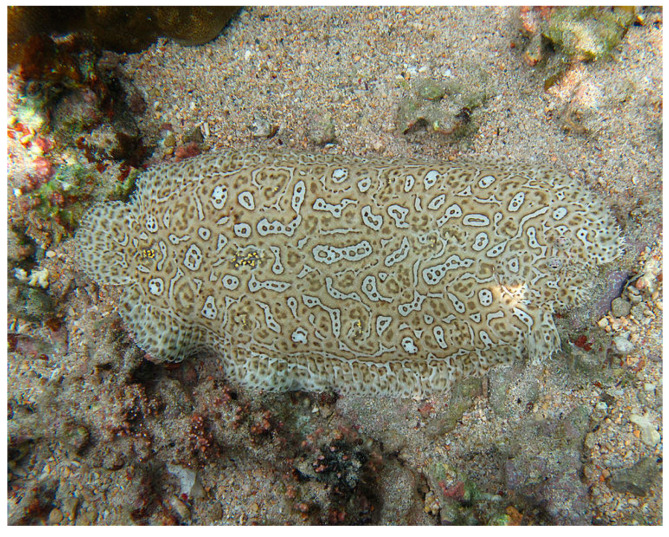
Specimen of *P. marmoratus*. Author: Philippe Bourjon (CC-BY-SA-4.0). https://commons.wikimedia.org/wiki/File:Pardachirus_marmoratus_%28Soleidae%29.jpg (accessed on 20 September 2023).

*Pardachirus marmoratus* (Lacepède, 1802; Chordata, Teleostei, Pleuronectiformes, Soleidae; Figure 30), also known as Red Sea Moses sole, or the finless sole or speckled sole, is a flatfish distributed in the western Indian Ocean and along the east coast of Africa. It is found in shallow coastal waters, where the bottom is sand or mud, near the coral reefs, where it feeds mainly on benthic invertebrates. This species shows a highly compressed body that is convex, with variable color, often whitish, tan, light gray with ring-shaped markings and dark brown spots on the head, body and fins. Important features are two brown spots, with two yellow spots, along the lateral line.

**Figure 31:**
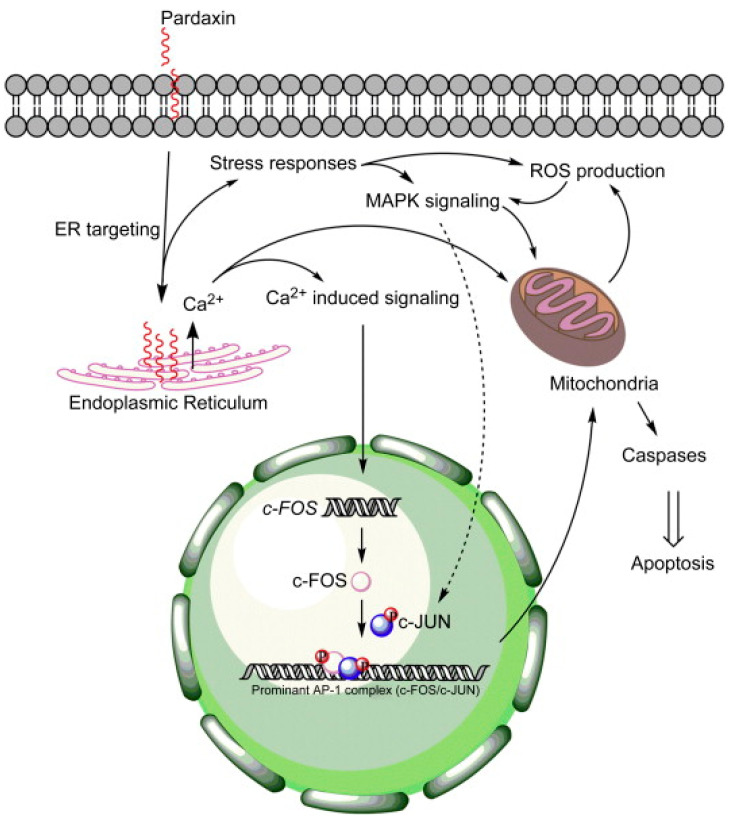
Proposed mechanism of action of pardaxin in fibrosarcoma cells. After cellular uptake, pardaxin selectively targets the endoplasmic reticulum, leading to Ca^++^ release and induction of *c-FOS* expression. Concurrently, an ROS-mediated stress response and MAPK signaling (e.g., ERK and JNK) contributes to mitochondrial dysfunction, activation of c-JUN/c-FOS complex and its downstream promoting effect on apoptosis [117].

**Figure 32:**
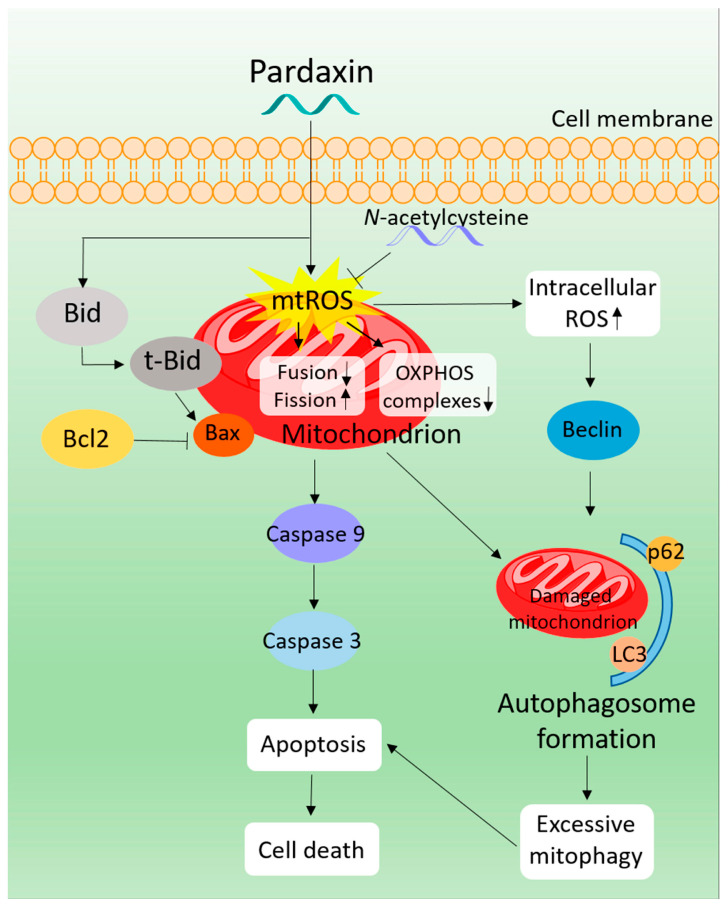
Proposed mechanism of action of pardaxin in ovarian cancer cells. After cellular uptake, pardaxin induces ROS overproduction (↑) in mitochondria, reinforced also by the attenuation of OXPHOS enzymatic complexes (↓), an imbalance in MMP, the up-regulation of t-Bid and Bax and activation of caspase-9 and -3 cascade, leading to the mitochondrial pathway of apoptosis. Mitochondrial fragmentation also occurs in parallel with autophagosome formation, thereby suggesting the activation of mitophagy [118] (CC-BY-4.0).

Pardaxin (HGFFALIPKISSPLFKTLSAVGSALSSSGGQE) is a cationic antimicrobial peptide isolated from the skin secretions of *P. marmoratus*. Hsu et al. [114] evaluated the antitumor activity of pardaxin against different tumor cells lines and found selective proliferation inhibition against human fibrosarcoma HT-1080 and cervical cancer HeLa cells. Pardaxin treatment induced significant lytic activity on the cellular or nuclear membranes of these tumor cells but, interestingly, not in red blood cells, consistent with the documented ability of the peptide to form stable or transient pores in zwitterionic lipid vesicles [115]. Moreover, pardaxin appeared able to selectively promote apoptosis on the sole HeLa cells, as evidenced by the DNA fragmentation, increased percentage of cells in the subG_1_ phase and the up-regulation of caspase-8 activity. Using proteomic approaches and network reconstruction, Huang and Chen [116] investigated the mechanism of pardaxin-induced apoptosis in HeLa cells. Their results highlighted the pardaxin-triggered production of ROS leading to oxidative stress and activation of the unfolded protein response. This, in turn, induced JNK/c-Jun and PERK/eIF2α/CHOP signalization, resulting in the onset of caspase- and apoptotic-inducing factor (AIF)-dependent apoptotic events, such as the loss of MMP, down-regulation of RhoGDI (conceivably orchestrating the initial morphology of apoptosis by controlling actin polymerization) and chromatin condensation. Ting et al. [117] used transcriptome analysis to screen for potential downstream targets of pardaxin, subsequently validating the obtained results through gene knockdown in an in vitro HT-1080 cell model system, and in in vivo tumor xenograft assays. As summarized in Figure 31, they showed that the death of fibrosarcoma cells was triggered by Ca^++^ signaling-stimulated induction of c-FOS, downstream to the direct pardaxin targeting to the endoplasmic reticulum. More recently, Chen et al. [118] evaluated the pardaxin effect on PA-1 and SKOV3 ovarian cancer cells (Figure 32). The peptide-induced mitochondria-mediated apoptosis caused by ROS overproduction in the organelle was paralleled by attenuation of OXPHOS enzymatic complexes, an imbalance in MMP, the up-regulation of t-Bid and Bax and activation of procaspase-9 and -3. In addition, microscopic analyses indicated that the mitochondrial network was fragmented, conceivably due to the down-regulation of the fusogenic protein, MFN1/2 and L-/S-OPA1, and the up-regulation of the fission-related proteins, DRP1 and FIS1. Autophagy was also activated as evidenced by the overexpression of the autophagosome formation-related proteins, Beclin, p62 and LC3. Enhanced mitochondrial fragmentation and autophagy suggested that mitophagy was activated.

**Figure 33:**
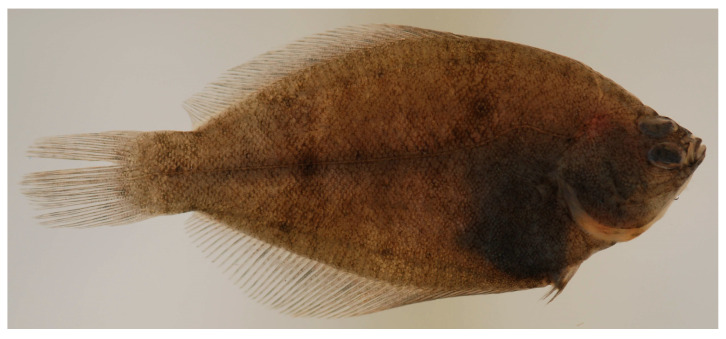
Specimen of *P. americanus*. Author: Smithsonian Environmental Research Center (CC-BY-2.0). https://commons.wikimedia.org/wiki/File:Pseudopleuronectes_americanus_%28S0892%29_%2812658628105%29.jpg (accessed on 16 January 2023).

*Pleuronectes americanus* (Walbaum, 1792; Chordata, Teleostei, Pleuronectiformes, Pleuronectidae; Figure 33), accepted to date as *Pseudopleuronectes americanus*, is a demersal and oceanodromous species distributed in the western Atlantic area. Adults of this species normally inhabit soft to moderately hard bottoms and feed mainly on organisms that live in, on or near the bottom, such as shrimps, amphipods, crabs, sea urchins and snails.

**Figure 34:**
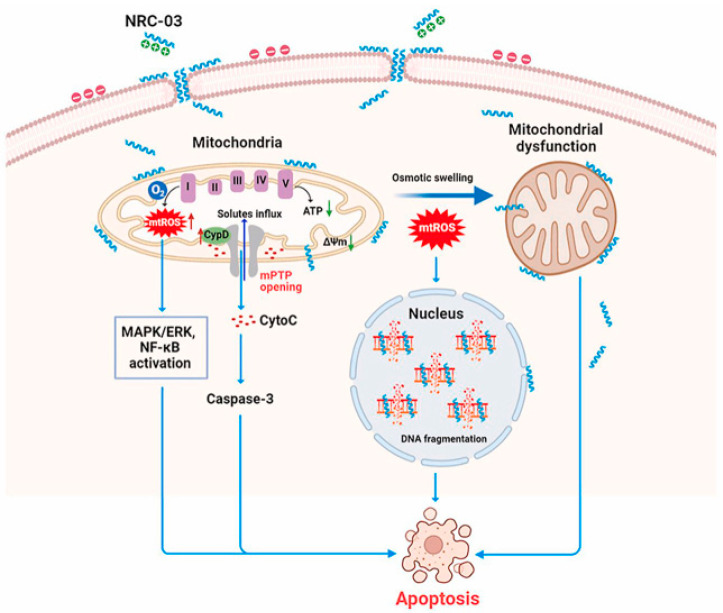
Proposed mechanism of action of NRC-03 on oral squamous cancer cells. The peptide targets the mitochondria and nucleus, causing mitochondria swelling, membrane blebbing and DNA fragmentation. ROS are produced in mitochondria via respiratory complex I (↑) in response to the increased oxygen consumption rate, and they activate MAPK/ERK and NF-κB signalization. NRC-03 also up-regulates cyclophilin D, thus stimulating mitochondrial pore opening and loss of transmembrane potential (↓ Δψm) that leads to the switching-on of apoptosis [121] (CC-BY-4.0).

Pleurocidins are a family of alpha-helical cationic antimicrobial peptides isolated from the skin mucous secretion of *P. americanus*. Hilchie et al. [119,120] reported that two members of the family, i.e., NRC-03 (GRRKRKWLRRIGKGVKIIGGAALDHL) and NRC-07 (RWGKWFKKATHVGKHVGKAALTAYL), were cytotoxic against various breast cancer cell lines, including drug-resistant variants, and multiple human myeloma cells, but not for human dermal fibroblasts, umbilical vein endothelial cells, normal peripheral blood mononuclear cells or erythrocytes. Exposure of breast cancer cells to the peptides led to the loss of MMP, as well as to the production of ROS, possibly as a result of the damage in mitochondria, conceivably resulting from pore formation. Interestingly, both NRC-03 and NRC-07 killed breast cancer cells grown in nonobese diabetic/severe combined immunodeficient (NOD/SCID) mice. Dealing with myeloma cells, the peptides (mostly NRC-03) induced pore formation in the plasmalemma and DNA fragmentation; moreover, intratumoral injections of NRC-03 impaired the development of multiple myeloma xenografts in immunocompromised mice. More recent studies have unveiled the mechanism of cytotoxicity of NRC-03 on CAL-27 and SCC-9 oral squamous cancer cells [121]. As shown in Figure 34, once the peptide enters the tumor cells it locates in the mitochondria and nucleus, causing membrane blebbing, mitochondria swelling and DNA cleavage. In particular, in mitochondria, it increases the oxygen consumption rate, causes ROS production via respiratory complex I, and activates MAPK/ERK and NF-κB signalization. In addition, NRC-03 up-regulates cyclophilin D, the key component of the mitochondrial permeability transition pore, thus stimulating pore opening that leads to the mitochondrial oxidative stress-mediated decrease in ATP production and the subsequent switching-on of apoptosis. Also, in this case, the intratumoral administration of the peptide inhibited the development of tumors in xenografted animal models.

## 5. Conclusions

It is widely acknowledged that the enormous biodiversity of marine organisms represents a highly promising reserve for the isolation of bioactive primary and secondary metabolites, targeting one or several specific molecular pathways and displaying active pharmacological properties against a variety of diseases. Among the anticancer compounds found in marine animals, in this review, a focus was put on peptides and their mechanisms of action in model systems in vitro. Cumulatively, anticancer peptides show several advantages compared to conventional chemotherapeutics, due to their low toxicity, biocompatibility, elevated specificity and selectivity and the low tendency to develop drug resistance [4]. Natural peptides have been modified in regard to their chemical structure, e.g., by methylation, acetylation or phosphorylation, as a strategy to improve their pharmacokinetic properties. It is noteworthy that many of them, apart from being “therapeutic”, have been used as “guiding missiles”, i.e., delivery carriers transporting poorly stable or insoluble drugs into the cancer cell targets, and “cell-stimulating drugs”, able to exert their anticancer effect indirectly, i.e., switching-on the host defense mechanism or switching-off hormone release [3,122,123]. On the other hand, it must also be considered that anticancer peptides have some potential drawbacks, such as a short half-life, low bioavailability, and production and manufacturing challenges [124]. In addition, chemically synthesized peptides have been applied in medicine, but their utilization has been severely limited due to their low systemic stability, high clearance, poor membrane permeability, negligible activity when administered orally and the high costs of manufacturing such products [125]. Anticancer peptides may also be buried in the structure of parental proteins and, therefore, must be obtained by enzymatic hydrolysis, but a caveat is that the final materials depend on the specific properties of the proteases used, the conditions of the proteolysis and the methods of sample recovery, all aspects that can compromise the bioactivity of the end product [126]. In conclusion, despite the extent of the marine environment’s biomedical chest, current research on this topic is still limited, and further study efforts are needed to expand the list of tested anticancer peptides discovered across the different taxonomic groups, as well as in vivo analyses and human trials, to ensure the effective chemotherapeutic efficacy of marine compounds as treatment options for different cancer histotypes.
